# Bi_2_S_3_/Ti_3_C_2_-TPP nano-heterostructures induced by near-infrared for photodynamic therapy combined with photothermal therapy on hypoxic tumors

**DOI:** 10.1186/s12951-024-02391-x

**Published:** 2024-03-20

**Authors:** Hanwen Jiang, Jingxian Sun, Fucong Liu, Yuanjiao Zhao, Xin Chen, Changsong Dai, Zhaohui Wen

**Affiliations:** 1 Department of Neurology, Brain Ultrasound, The First Affiliated Hospital of Harbin Medical University, Harbin, Heilongjiang Province 150001 China; 2 Department of Cardiology, Cardiac Ultrasound, The First Affiliated Hospital of Harbin Medical University, Harbin, Heilongjiang Province 150001 China; 3 Key Colleges and Universities Laboratory of Neurosurgery in Heilongjiang Province, Harbin, Heilongjiang Province 150001 China; 4 MIIT Key Laboratory of Critical Materials Technology for New Energy Conversion and Storage, School of Chemistry and Chemical Engineering, Harbin Institute of Technology, Harbin, 150001 China; 5 Department of Neurosurgery, The Affiliated Hospital of Qingdao University, Qingdao, Shandong Province 266005 China

**Keywords:** Nano-heterostructure, Photothermal therapy, Photodynamic therapy, Hypoxic tumor, Tumor theranostics

## Abstract

**Background:**

Photodynamic therapy (PDT) efficacy of bismuth sulfide (Bi_2_S_3_) semiconductor has been severely restricted by its electron–hole pairs (e^−^−h^+^) separation inefficiency and oxygen (O_2_) deficiency in tumors, which greatly hinders reactive oxygen species (ROS) generation and further clinical application of Bi_2_S_3_ nanoparticles (NPs) in biomedicine.

**Results:**

Herein, novel Bi_2_S_3_/titanium carbide (Ti_3_C_2_) two-dimensional nano-heterostructures (NHs) are designed to realize multimode PDT of synchronous O_2_ self-supply and ROS generation combined with highly efficient photothermal tumor elimination for hypoxic tumor therapy. Bi_2_S_3_/Ti_3_C_2_ NHs were synthesized via the in situ synthesis method starting from Ti_3_C_2_ nanosheets (NSs), a classical type of MXene nanostructure. Compared to simple Bi_2_S_3_ NPs, Bi_2_S_3_/Ti_3_C_2_ NHs significantly extend the absorption to the near-infrared (NIR) region and enhance the photocatalytic activity owing to the improved photogenerated carrier separation, where the hole on the valence band (VB) of Bi_2_S_3_ can react with water to supply O_2_ for the electron on the Ti_3_C_2_ NSs to generate ·O_2_^−^ and ·OH through electron transfer. Furthermore, they also achieve ^1^O_2_ generation through energy transfer due to O_2_ self-supply. After the modification of triphenylphosphium bromide (TPP) on Bi_2_S_3_/Ti_3_C_2_ NHs, systematic in vitro and in vivo evaluations were conducted, revealing that the synergistic-therapeutic outcome of this nanoplatform enables complete eradication of the U251 tumors without recurrence by NIR laser irradiation, and it can be used for computed tomography (CT) imaging because of the strong X-ray attenuation ability.

**Conclusion:**

This work expands the phototherapeutic effect of Bi_2_S_3_-based nanoplatforms, providing a new strategy for hypoxic tumor theranostics.

**Supplementary Information:**

The online version contains supplementary material available at 10.1186/s12951-024-02391-x.

## Introduction

In recent years, nanomaterial (NM)-mediated phototherapy, including photodynamic therapy (PDT) and photothermal therapy (PTT), has shown great potential as an emerging method for oncotherapy, owing to its advantages of minimal invasiveness, regional selectivity, negligible toxic and side effects, short treatment time, and repeatable treatments [1]. At present, numerous NMs have been developed for PTT [2], but the progress of PDT is restricted due to the excitation of most photosensitizers (PSs) only by ultraviolet and visible light, the low electron–hole pair (e^−^−h^+^) separation efficiency, and the reduced therapeutic effect caused by the massive consumption of tissue oxygen (O_2_) during the treatment process [3]. Besides, tumor tissue inherently presents a hypoxic tumor microenvironment (TME) because of the distortional blood vessels, the rapid proliferation, and the exuberant metabolism of tumor cells [4]. In order to enhance the poor PDT effect attributed to hypoxia, various techniques of O_2_ carrying and catalytic production of O_2_ were investigated and applied [5], but the asynchronism between O_2_ supply and reactive oxygen species (ROS) formation made the therapeutic effect on tumors not ideal. Although PTT is a non-O_2_-dependent therapeutic method [6], if photothermal agents (PTAs) are not uniformly dispersed in the distorted tumor vessels during treatment, the temperature rise would be uneven, which may also weaken the oncotherapy efficacy [7]. As a result, developing a simple nanoplatform that combines both PDT and PTT functions under near-infrared (NIR) irradiation at the same wavelength to achieve a synergistic therapeutic effect with the generation of ROS and heat while producing O_2_ has greater clinical potential. Such a nanoplatform would have stronger ROS generation capacity and better therapeutic efficacy than nanomaterials known to have both PTT and PDT functions (e.g., indocyanine green), owing to its excellent oxygen supply capacity [8].

The ability of PSs to generate both ROS and O_2_ simultaneously depends on their unique internal electronic structure. Photosensitive semiconductor NMs with relatively narrow bandgaps can be excited by NIR at 800-1300 nm to make the photoinduced electrons in the valence band (VB) migrate to the conduction band (CB), leaving positive holes [9]. These free-moving photoinduced electrons and holes with redox abilities can, respectively, react with the surrounding O_2_ and water molecules under certain potential conditions to produce the killing superoxide anion (·O_2_^-^) radicals and O_2_ [10]. Moreover, as a primary ROS, ·O_2_^-^ can be further transformed into a series of ROS with stronger tumor-killing property, such as hydrogen peroxide (H_2_O_2_) and hydroxyl (·OH) radicals [11]. ·O_2_^-^ radicals are produced through the type I PDT mechanism by the electron transfer pathway, whereas most PSs are based on the type II PDT mechanism, in which energy transfers to O_2_ in adjacent tissues to generate toxic singlet oxygen (^1^O_2_) [12]. Bismuth sulfide (Bi_2_S_3_) nanoparticles (NPs) have excellent optical catalytic activity and a high NIR absorption coefficient, and have been widely utilized as PTAs [13] and computed tomography (CT) contrast agents [14] in recent decades to realize the integration of multi-modal diagnosis and treatment of tumors [15, 16], but their direct application in PDT is limited due to the narrow bandgap. Nevertheless, Bi_2_S_3_-based nano-heterostructures (NHs) with appropriate wavelength absorption and boosted photogenerated carrier separation efficiency are promising for establishing PSs with novel properties. Regrettably, there has been little research in this field.

**Scheme 1:**
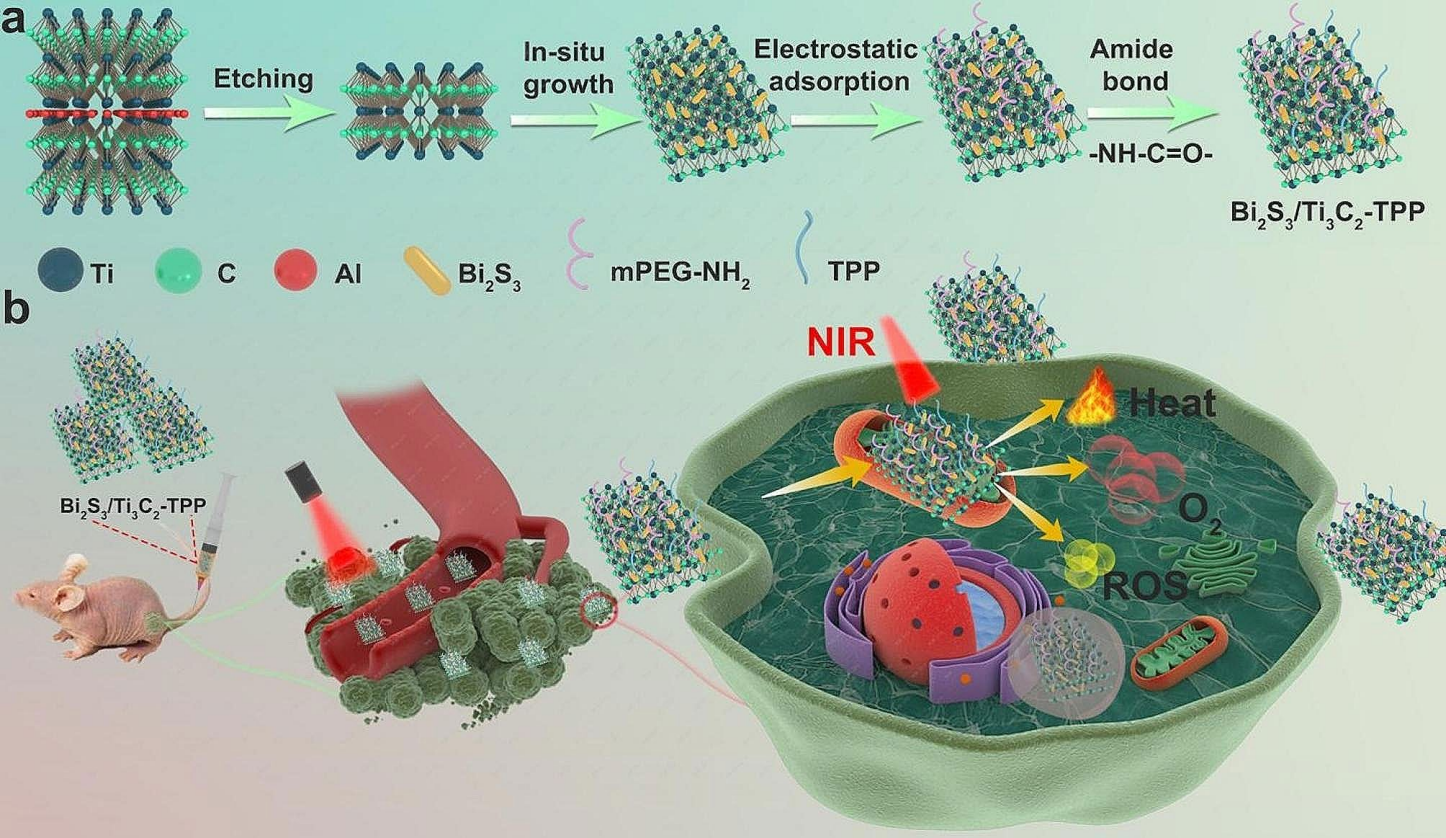
Schematic Illustration of the Fabrication Process and Therapeutic Mechanism of Bi_2_S_3_/Ti_3_C_2_-TPP. **a** Bi_2_S_3_/Ti_3_C_2_-TPP was prepared by *in-situ* growth of Bi_2_S_3_ NPs in Ti_3_C_2_ NSs and modification with TPP. **b** Bi_2_S_3_/Ti_3_C_2_-TPP exerted a synergistic therapeutic effect of mitochondria-targeted multi-mode PDT combined with PTT on hypoxic tumors under NIR irradiation

Herein, we designed a new nanoplatform, Bi_2_S_3_/Ti_3_C_2_-TPP (TPP, triphenylphosphium bromide), that was synthesized via the in situ synthesis method starting from titanium carbide (Ti_3_C_2_), two-dimensional ultra-thin ceramic nanosheets (NSs) with satisfactory photothermal conversion efficiency (PTCE,𝜂), excellent electrical features, and a large specific surface area [17], then further attached with TPP on the surface (Scheme 1). Compared to simple Bi_2_S_3_ NPs, Bi_2_S_3_/Ti_3_C_2_-TPP significantly extends absorption to the NIR region, enhances photocatalytic activity due to higher photogenerated carrier separation and electron transfer efficiency, and can also effectively aggregate in tumor cells by targeting the mitochondria showing extraordinary CT imaging capability. Under 808 nm laser irradiation, Bi_2_S_3_ NPs generate free-moving holes in the VB and electrons in their CB that migrate to Ti_3_C_2_ NSs connected closely with Bi_2_S_3_ [18], during which ·O_2_^-^ and ·OH were produced via the type I PDT mechanism by electron transfer and ^1^O_2_ was generated via the type II PDT mechanism by energy transfer. Therefore, Bi_2_S_3_/Ti_3_C_2_-TPP demonstrated powerful tumor diagnosis and treatment functions in both normoxic and hypoxic TME.

## Materials and methods

### Materials

HCl (36–38%), LiF, Ti_3_AlC_2_ (325-meshes), and TPAOH were supplied by Beijing Chemical Reagents Company (Beijing, China), Aladdin Bio-Chem Technology Corporation (Shanghai, China), Forsman Scientific Company (Beijing, China), and J&K Scientific Corporation (Beijing, China), respectively. Oleic acid (OA), oleylamine, thioacetamide, bismuth neodecanoate, plysorbate 20 (Tween 20), cyclohexane, mPEG-NH_2_ (Mw$\approx$2K), N-(3-dimethylaminopropyl)-N’ethylcarbodiimide hydrochloride (EDC), N-hydroxysuccinimide (NHS), TPP, dimethyl sulfoxide (DMSO), 5,5-dimethyl-1-pyrroline N-oxide (DMPO), 2,2,6,6-tetramethylpiperidine (TEMP), CPZ, and colchicine were purchased from Sigma-Aldrich (MO, USA). MTT, DCFH-DA, mitochondrial membrane potential assay kit (JC-1), cell lysis buffer (RIPA), Annexin V-FITC/propidium iodide (PI) apoptosis and necrosis detection kit, H&E staining kit, and TUNEL apoptosis assay kit were obtained from Beyotime Institute of Biotechnology (Shanghai, China). Calcein Acetoxymethyl ester (Calcein-AM)/PI double stain kit was brought from Yeasen Biotechnology Corporation (Shanghai, China). PBS (pH = 7.4, 10 mM), fetal bovine serum (FBS), trypsin, penicillin-streptomycin (PS), reduced serum medium (Opti-MEM), DMEM, mitochondrion-selective probe (Mito-Tracker Red), and red hypoxia probe (Image-iT™) were provided by Gibco Life Technologies (CA, USA). All the polyclonal antibodies were obtained from Proteintech Group Incorporation (IL, USA). All chemicals were used as received without further purification.

### Synthesis of Bi_2_S_3_/Ti_3_C_2_

Ti_3_C_2_ NSs were prepared in a typical way of etching the Al atomic layer from Ti_3_AlC_2_ with HCl/LiF. Briefly, LiF (5 g) was dissolved in a HCl aqueous solution (50 mL, 9 mmol) contained in a polytetrafluoroethylene (PTEF) beaker and magnetically stirred for 30 min at room temperature to acquire a hydrogen fluoride (HF) aqueous solution. HF and HCl are both highly corrosive liquids, and the experimenters must strictly follow the safety protocol during the experiment. Then, Ti_3_AlC_2_ powders (5 g) were slowly added in the HF aqueous solution and stirred at $38^\circ C$ for 3 days. After being washed and centrifuged repeatedly with deionized water (DI) or ethanol (11,000 rpm $\times$ 10 min) to make the supernatant pH close to 7, the collected precipitates were dispersed in 25 mL TPAOH with stirring at room temperature for 3 days. The resulting ultra-thin Ti_3_C_2_ NSs were collected by freeze-drying after washing and centrifugation with DI or ethanol several times (11,000 rpm $\times$ 10 min). Particularly, Bi_2_S_3_/Ti_3_C_2_ NHs were fabricated by a solvothermal synthetic method in situ. Initially, Ti_3_C_2_ NSs (0.25 g) were evenly dispersed in ethanol (10 mL) with an OA (20 mL) addition, and bismuth neodecanoate (1.45 g) was added to the mixed solution under continuous agitation, which made the bismuth ion (Bi^3+^) attached to the Ti_3_C_2_ NSs by electrostatic absorption. Subsequently, an oleylamine solution (4 mL) with thioacetamide (0.15 g) dissolved in it was rapidly added to the above solution with violent stirring at room temperature for 1 h. Eventually, the prepared mixture was sealed in a Teflon-lined autoclave (50 mL) and heated at 150 $^\circ C$ for 10 h, which was required to cool to room temperature naturally at the end of the reaction. After being washed and centrifuged with ethanol (10,000 rpm $\times$ 5 min) three times, the oil-soluble OA-coated Bi_2_S_3_/Ti_3_C_2_ NHs were obtained by freeze-drying. Similarly, Bi_2_S_3_ NPs were fabricated without the addition of Ti_3_C_2_ NSs.

### Fabrication of Bi_2_S_3_/Ti_3_C_2_-TPP

In order to connect TPP to Bi_2_S_3_/Ti_3_C_2_ NHs, oil-soluble OA-coated Bi_2_S_3_/Ti_3_C_2_ NHs must be converted to water-soluble NHs and encapsulated with mPEG-NH_2_. Firstly, OA-coated Bi_2_S_3_/Ti_3_C_2_ NHs (100 mg) and Tween 20 (150 $\mu$L) were uniformly dispersed in cyclohexane (20 mL) with stirring at room temperature for 1.5 h. After that, the mixture was added dropwise into DI (30 mL) in a 70 $^\circ C$ water bath and continuously stirred for at least 3 h to evaporate the cyclohexane. Later, the water-soluble Tween 20-functionalized Bi_2_S_3_/Ti_3_C_2_ NHs were collected by freeze-drying after washing and centrifugation with ethanol (10,000 rpm $\times$ 5 min) three times. Then, mPEG-NH_2_ (20 mg) and Tween 20-functionalized Bi_2_S_3_/Ti_3_C_2_ NHs (20 mg) were sufficiently dispersed in DI (10 mL) and stirred overnight at room temperature, while EDC (50 mg) and NHS (25 mg) were added into an aqueous solution (10 mL) containing TPP (50 mg) and stirred in the dark at room temperature for 6 h to activate TPP. Finally, the two prepared solutions were mixed with stirring overnight in the dark, and Bi_2_S_3_/Ti_3_C_2_-TPP was obtained by freeze-drying after washing and centrifugation with DI (10,000 rpm $\times$ 5 min) three times.

### Characterization

SEM (1000000X, TESCAN, CZ), TEM (JEM-2100, JEOL, JPN), and AFM (Dimension Fastscan, BRUKER, GER) were used to describe the morphology and structure of the samples. XRD (X’PERT, Panalytical, NL), XPS (ESCALAB 250Xi, ThermoFisher, USA), and FT-IR (Nicolet is50, ThermoFisher, USA) were conducted to determine the composition of the samples. ICP-MS, PL, UV-vis, and ESR spectroscopy were collected on an iCAP7400 spectrometer (ThermoFisher, USA), a FLS1000 spectrophotometer (Edinburgh, UK), a LAMBDA950 spectrophotometer (PE, USA), and an A200S-95/12 spectrometer (BRUKER, GER), respectively. The average size and zeta potential of the samples were measured by Nano-MS2000/ZS90 (Malvern, UK). The NIR source was purchased from LeiShi (808 nm, China). Oxygen concentration and temperature were severally monitored by a dissolved oxygen meter (JPBJ-607 A, INESA, China) and a portable thermal imager (E5-XT, FLIR, USA). All fluorescent imaging was visualized by an inverted fluorescence microscope (AF6000, Leica, GER). Cell viability was evaluated by a microplate reader (ELX800, Bio Tek, USA), while a flow cytometer (NovoCyte, Agilent, USA) was used to conduct quantitative flow cytometry. Western blot exposure was performed on a chemiluminescence imager (ChemiDoc Touch, Bio-Rad, USA).

### Photodynamic performance and O_2_ evolution evaluation

ROS assay kits were used to detect the ROS generation capacity of Bi_2_S_3_/Ti_3_C_2_, Ti_3_C_2_, and Bi_2_S_3_ under 808 nm laser irradiation. Initially, the DCFH-DA was diluted with ethanol to acquire a solution with a molar concentration of 1 mM, and the mixture (0.5 mL) was added to the NaOH solution (2 mL, 10 mM) with stirring at room temperature for 30 min in the dark. After that, the obtained mixed solution was uniformly dispersed in PBS (10 mL, pH = 7.4, 10 mM) and stored at 4 $^\circ C$ in a refrigerator. Then, the prepared DCFH-DA solution (100 $\mu$L) was mixed with the sample solution (100 $\mu$L, 200 $\mu$g mL^-1^) and irradiated with an 808 nm laser (1 W cm^-2^, 10 min). Meanwhile, a PL spectrophotometer was used to measure the fluorescence intensity of DCF per minute in order to determine how ROS generation changed over time.

ESR measurements were performed to identify the ROS species generated by Bi_2_S_3_/Ti_3_C_2_ under NIR irradiation. Briefly, TEMP as a spin-trapping agent was added to the methanol solution of Bi_2_S_3_/Ti_3_C_2_ (500 $\mu$L, 100 $\mu$g mL^-1^) to reach a molar concentration of 200 $\mu$mol L^-1^, and the mixture was irradiated with an 808 nm laser (1 W cm^-2^, 10 min). Whereafter, an ESR measurement was immediately conducted to determine whether ^1^O_2_ was formed. The formation of ·OH and ·O_2_^-^ was tested by the same experimental procedure in DI and methanol, respectively, and DMPO was used as the spin-trapping agent.

Nitrogen (N_2_) was blown into the Bi_2_S_3_/Ti_3_C_2_ solution (10 mL, 100 $\mu$g mL^-1^) for 15 min to remove the inherent O_2_ in it, and the solution was sealed with liquid paraffin. After that, the amount of O_2_ produced by the samples under 808 nm laser irradiation (1 W cm^-2^) was measured per minute within 20 min, utilizing a dissolved oxygen meter.

### Photothermal performance evaluation

To evaluate the photothermal effect of the samples, Bi_2_S_3_/Ti_3_C_2_, Ti_3_C_2_, or Bi_2_S_3_ aqueous solutions (4.5 mL, 100 $\mu$g mL^-1^) were added to a tailor-made quartz cuvette and irradiated with an 808 nm laser (1 W cm^-2^, 10 min). A thermal imager was used to record temperature changes every 30 s, until it reached room temperature approximately. Simultaneously, the above experimental procedure was continuously repeated five times to further investigate the photothermal stability of Bi_2_S_3_/Ti_3_C_2_. Subsequently, the temperature changes of Bi_2_S_3_/Ti_3_C_2_ aqueous solutions with different concentrations (0, 25, 50, 100, and 200 $\mu$g mL^-1^) were recorded within 10 min under the same conditions, aiming to determine the influence of sample concentration on the photothermal performance.

### Enrichment analysis of hypoxic-related genes in glioma

Firstly, gene expression and clinical data from glioma samples were gathered through the TCGA and CGGA databases. Then, the hypoxic-related gene set was chosen from The Molecular Signatures Database (MSigDB, http://www.gsea-msigdb.org/gsea/msigdb/human/search.jsp). The samples were classified into low-grade glioma and glioblastoma based on tumor grade, and the enrichment degree of hypoxic-related genes in the two samples was determined by GSEA. Whereafter, the R package of gene set variation analysis (GSVA) and ssGSEA were used to acquire hypoxic-related functional scores for each glioma patient sample in the TCGA and CGGA databases. Finally, the scores were analyzed in conjunction with tumor grade and survival time to explore their relationship.

### Cellular experiments

U251 human glioma cells were selected for the following cellular investigations and cultured according to the standard protocol. For cell culture in a hypoxic microenvironment, the conditions were set as a humidified atmosphere containing 1% O_2_ and 5% CO_2_ at 37 $^\circ C$, which was formed by flowing N_2_.

For cellular uptake, 8 $\times$ 10^4^ U251 cells in 1 mL of DMEM were seeded in each well of a 12-well plate for 24 h of incubation. Then, cells were treated with Opti-MEM containing Bi_2_S_3_/Ti_3_C_2_-TPP or Bi_2_S_3_/Ti_3_C_2_ (12.5, 25, 50, 100, or 200 $\mu$g mL^-1^) for 4 h. After washing with PBS three times, cells were digested with trypsin and collected by centrifugation (1000 rpm $\times$ 5 min, 4 $^\circ C$). Finally, cells were counted, and the Bi or Ti content was measured by ICP-MS tests.

For the hemolysis assay, 0.5 mL of blood was obtained from mice, and erythrocytes were extracted by centrifugating and washing with PBS five times (3000 rpm $\times$ 5 min, 4 $^\circ C$) and diluted at 10 times their initial volume in PBS. Then, diluted suspensions (200 $\mu$L) were mixed with 800 $\mu$L of PBS, Triton-100 (0.025% in PBS), and Bi_2_S_3_/Ti_3_C_2_-TPP (25, 50, 100, 200, and 300 $\mu$g mL^-1^ in PBS) and incubated with cells at 37 $^\circ C$ for 2 h. All samples were centrifuged (3000 rpm $\times$ 5 min, 4 $^\circ C$), and the supernatant absorbance at 541 nm was detected using a microplate reader. The hemolysis rate was calculated by dividing the supernatant absorbance value (I) of the samples by the value (I_0_) of the positive control group. The calculation formula is as follows:

Hemolysis rate (%) = (I/I_0_) $$\times$$ 100%

For mitochondrial targeting performance, 1.6 $\times$ 10^5^ U251 cells in 2 mL DMEM were seeded in dishes (d = 35 mm) for 24 h of incubation. After treatment with Opti-MEM containing Bi_2_S_3_/Ti_3_C_2_-TPP (100 $\mu$g mL^-1^) for 4 h, Mito-Tracker Red (1 $\mu$L, 400 nmol L^-1^) was added to the medium, and cells were incubated for another 15 min. Finally, cells were washed with PBS three times and imaged with an inverted fluorescence microscope.

For mitochondrial membrane potential assessment, 8 $\times$ 10^4^ U251 cells in 1 mL of DMEM were seeded in each well of a 12-well plate for 24 h of incubation. After treatment with Opti-MEM containing different NMs (100 $\mu$g mL^-1^) for 4 h, cells were irradiated with or without an 808 nm laser (1 W cm^-2^, 10 min). After another 2 h of incubation, cells were stained with JC-1 (5 $\mu$mol L^-1^, 20 min) in Opti-MEM for mitochondrial membrane potential. Finally, cells were washed with PBS three times and directly observed by an inverted fluorescence microscope or quantitatively analyzed by flow cytometry.

For intracellular O_2_ detection, 1 $\times$ 10^4^ U251 cells in 100 $\mu$L of DMEM were seeded in each well of a 96-well plate for 24 h of incubation. After treatment with Opti-MEM containing Bi_2_S_3_/Ti_3_C_2_-TPP (100 $\mu$g mL^-1^) for 4 h, Image-iT™ was added to the medium to a concentration of 10 $\mu$mol L^-1^, and cells were incubated for another 30 min. Then, the medium was renewed, and cells were incubated in a hypoxic culture environment for 4 h. After the incubation, cells were irradiated with or without an 808 nm laser (1 W cm^-2^, 10 min) and imaged with an inverted fluorescence microscope.

For intracellular ROS detection, 8 $\times$ 10^4^ U251 cells in 1 mL of DMEM were seeded in each well of a 12-well plate for 24 h of incubation. After treatment with Opti-MEM containing Bi_2_S_3_/Ti_3_C_2_-TPP (100 $\mu$g mL^-1^) for 4 h, cells were stained with DCFH-DA (10 $\mu$mol L^-1^) in Opti-MEM for 15 min and irradiated with or without an 808 nm laser (1 W cm^-2^, 10 min). Finally, the cellular DCF fluorescence was directly observed by an inverted fluorescence microscope or quantitatively analyzed by flow cytometry after washing with PBS three times.

For in vitro phototherapy, 1 $\times$ 10^4^ U251 cells in 100 $\mu$L DMEM were seeded in each well of a 96-well plate for 24 h of incubation. After treatment with Opti-MEM containing different NMs (100 $\mu$g mL^-1^) for 4 h, cells were irradiated with or without an 808 nm laser (1 W cm^-2^, 10 min) and incubated for another 20 h. Then, cell viability was evaluated by MTT and live/dead staining assays. For PDT fractions, the temperature during NIR irradiation should be 4 $^\circ C$ to avoid the influence of PTT.

For the cell clone formation assay, 1 $\times$ 10^3^ U251 cells in 2 mL DMEM were seeded in each well of a 6-well plate for 2 d of incubation. After treatment with Opti-MEM containing different NMs (100 $\mu$g mL^-1^) for 4 h, cells were irradiated with or without an 808 nm laser (1 W cm^-2^, 10 min) and incubated for another 10 d with medium changed every other day. When significant clone clusters were visible under the microscope, the incubation was terminated. Then, cells were washed with PBS and fixed with formaldehyde for 15 min. Finally, 0.1% crystal violet was added to the samples to stain for 10 min, which was photographed for recording.

For the cell apoptosis assay, 1.6 $\times$ 10^5^ U251 cells in 2 mL DMEM were seeded in each well of a 6-well plate for 24 h of incubation. After treatment with Opti-MEM containing different NMs (100 $\mu$g mL^-1^) for 4 h, cells were irradiated with or without an 808 nm laser (1 W cm^-2^, 10 min) and incubated for another 20 h. Then, cells were washed with cold PBS, collected, and stained with the Annexin V-FITC/PI assay kit. Finally, they were detected by flow cytometry.

For Nrf2, HO-1, and Hsp70 expressions, 1.6 $\times$ 10^5^ U251 cells in 2 mL DMEM were seeded in each well of a 6-well plate for 24 h of incubation. After treatment with Opti-MEM containing different NMs (100 $\mu$g mL^-1^) for 4 h, cells were irradiated with or without an 808 nm laser (1 W cm^-2^, 10 min) and incubated for another 20 h. Then, cells were washed with cold PBS and collected. The protein content was determined by the Bradford method, and the expressions of Nrf2, HO-1, Hsp70, and β-actin were evaluated through western blotting analysis.

### Animal experiments

Four-week-old female Balb/c nude mice were purchased from Liaoning Changsheng Biotechnology Co., Ltd., and all animal experiments were approved by the Animal Care and Ethical Committee of the First Affiliated Hospital of Harbin Medical University, which were conducted in accordance with the guidelines from the Ministry of Science and Technology of China.

For in vivo phototherapy, the U251 tumor model was established by subcutaneous injection of PBS (100 $\mu$L) containing 1 $\times$ 10^6^ U251 cells into the right back of the hips of each mouse. When the tumor volume of mice reached approximately 80 mm^3^ (V = width^2^ $\times$ length $\times$ 1/2) under the same feeding conditions, total mice were randomly divided into 7 treatment groups (*n* = 5) and injected through the caudal vein with (I) PBS (pH = 7.4, 10 mM); (II) PBS (pH = 7.4, 10 mM) with an 808 nm laser irradiation (1 W cm^-2^, 10 min); (III) Bi_2_S_3_/Ti_3_C_2_-TPP; (IV) Bi_2_S_3_ with an 808 nm laser irradiation (1 W cm^-2^, 10 min); (V) Bi_2_S_3_ + Ti_3_C_2_ with an 808 nm laser irradiation (1 W cm^-2^, 10 min); (VI) Bi_2_S_3_/Ti_3_C_2_ with an 808 nm laser irradiation (1 W cm^-2^, 10 min); (VII) Bi_2_S_3_/Ti_3_C_2_-TPP with an 808 nm laser irradiation (1 W cm^-2^, 10 min), respectively. The injection concentration of the samples contained in PBS (200 $\mu$L) was 20 mg kg^-1^, with a Bi_2_S_3_ to Ti_3_C_2_ ratio of 3:2 in Group V. And the samples were irradiated at 24 h post-injection. Under NIR irradiation, the temperature of tumor sites in mice was recorded by a portable thermal imager. After treatments, the tumor volumes and body weights of mice were monitored every other day during 2 weeks. Furthermore, the mice were euthanized after 2-week tumor treatment, and the tumor tissues and major organs (heart, liver, spleen, lung, and kidney) of mice in each group were harvested, sliced, and stained for H&E and TUNEL staining to perform the histological analysis.

For in vivo biodistribution and optical imaging assessment, U251 tumor-bearing mice were intravenously injected with Bi_2_S_3_/Ti_3_C_2_-TPP (20 mg kg^-1^). At indicated time points (1, 2, 4, 8, 12, and 24 h post-injection), 50 $\mu$L blood was extracted from the caudal vein of each mouse and weighed every time (*n* = 5 for each time point). The mice were sacrificed after the last blood collection, and tumors and major organs were harvested. Then, the content of Bi or Ti in the blood and tissue samples was measured by ICP-MS tests. In vivo optical imaging was presented by CT imaging. The mouse was anesthetized at 0 and 8 h post-injection, and imaged by a CT scanner. Then, the CT value of the region of interest (ROI) at two time points was evaluated and compared with the corresponding analysis software.

### Statistical analysis

SPSS 23.0 statistical software was conducted for the statistical analysis of the experimental data. A completely random design was performed to obtain measurement data. The normal distribution was described by mean $\pm$ standard deviation, and the non-normal distribution was described by quartile and median. The analysis of variance (ANOVA) was used for comparison between multiple groups, and the *t* test was used for comparison between two groups. *P* $<$ 0.05 was considered statistically significant.

## Results and discussion

### Fabrication and characterization of Bi_2_S_3_/Ti_3_C_2_-TPP

**Fig. 1:**
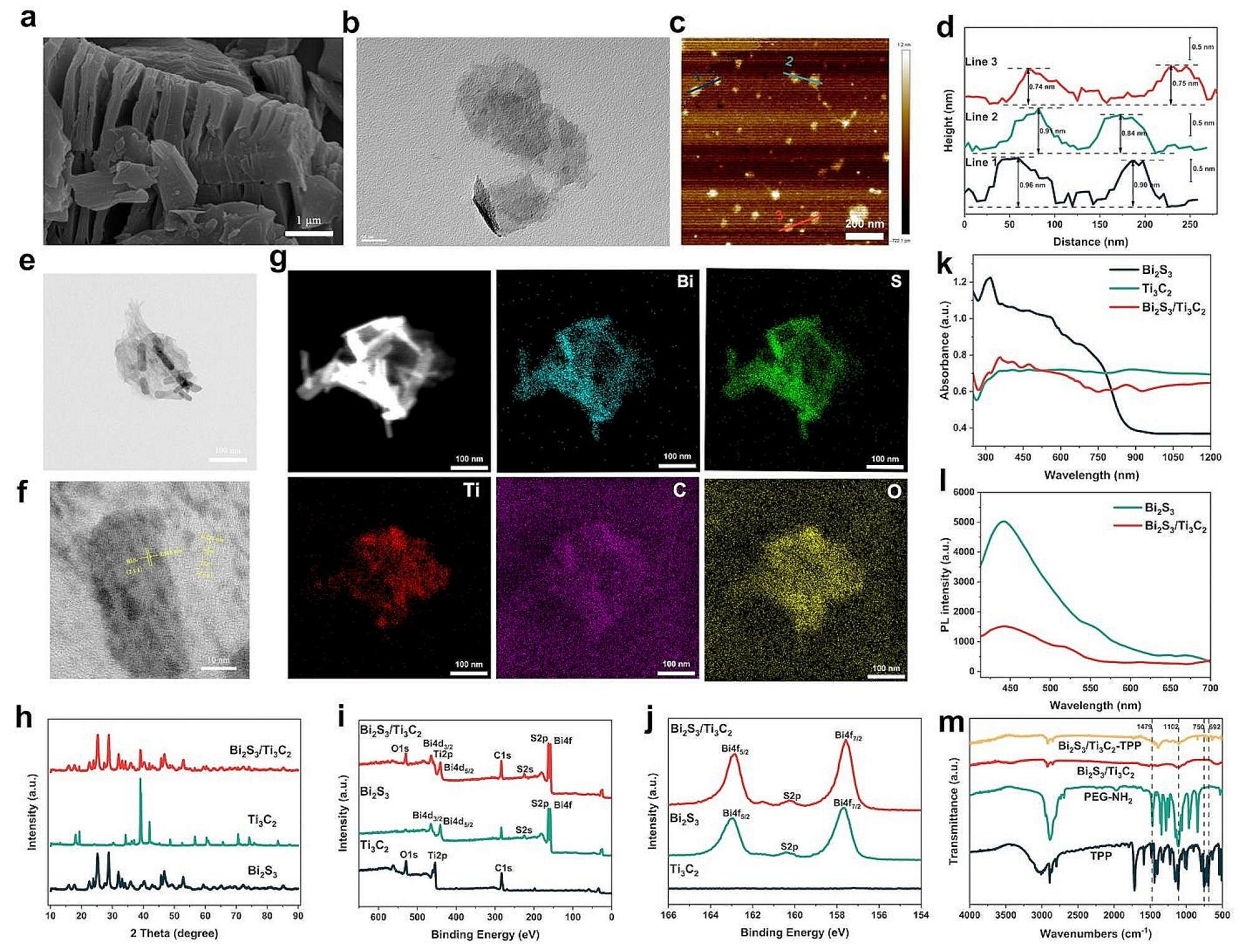
Fabrication and characterization of Bi_2_S_3_/Ti_3_C_2_-TPP. **a** SEM image of Ti_3_C_2_ powders. **b** TEM image of Ti_3_C_2_ NSs. **c-d** AFM images of Ti_3_C_2_ NSs. **e** TEM image, **f** HRTEM image, and **g** EDS mapping images (Bi-blue, S-green, Ti-red, C-purple, and O-yellow) of Bi_2_S_3_/Ti_3_C_2_ NHs. **h** XRD patterns, **i-j** XPS spectra, and **k** UV-vis absorption spectra of Bi_2_S_3_ NPs, Ti_3_C_2_ NSs, and Bi_2_S_3_/Ti_3_C_2_ NHs. **l** PL spectra of Bi_2_S_3_ NPs and Bi_2_S_3_/Ti_3_C_2_ NHs. **m** FT-IR patterns of TPP, PEG-NH_2_, Bi_2_S_3_/Ti_3_C_2_, and Bi_2_S_3_/Ti_3_C_2_-TPP

Two-dimensional Ti_3_C_2_ NSs were synthesized by the typical method of liquid-phase chemical etching and exfoliation described in the previous literature [19, 20]. Firstly, titanium aluminum carbide (Ti_3_AlC_2_) powder, the MAX phase precursor, was etched with a relatively mild mixed aqueous solution of lithium fluoride (LiF) and hydrogen chloride (HCl) to remove the Al layers, yielding well-stacked and uniform-thickness multilayer Ti_3_C_2_ sheets. Then, in order to acquire ultrathin Ti_3_C_2_ NSs, the multilayer Ti_3_C_2_ sheets were further delaminated by tetrapropylammonium hydroxide (TPAOH) intercalation to increase layer spacing and adequately dispersed by ultrasound to reduce their planar sizes. The microstructures of multilayer massive Ti_3_C_2_ formed after the etching of Ti_3_AlC_2_ powder and ultrathin Ti_3_C_2_ NSs obtained after exfoliation and ultrasonic dispersion were clearly visible in scanning electron microscopy (SEM), transmission electron microscopy (TEM), and atomic force microscopy (AFM) images. According to the SEM image, Ti_3_C_2_ sheets had multilayer stacked structures with uniform thickness (Fig. 1a). The TEM image showed that the average planar size of virtually transparent ultrathin Ti_3_C_2_ NSs was about 100 nm (Fig. 1b), while the AFM images indicated that the mean thickness was about 1 nm (Fig. 1c, d). Bi_2_S_3_ NPs were synthesized in situ on the surface of ultrathin Ti_3_C_2_ NSs by the solvothermal synthesis method to obtain oil-soluble Bi_2_S_3_/Ti_3_C_2_ NHs [21, 22]. The TEM image revealed that Bi_2_S_3_ NPs with approximately 65 nm in length and 15 nm in diameter were randomly loaded on the exterior of Ti_3_C_2_ NSs (Fig. 1e), and two fringes with interplanar distances of 0.310 nm and 0.231 nm were matched with the (2 1 1) plane of Bi_2_S_3_ and (1 0 4) plane of Ti_3_C_2_, respectively, according to the high-resolution TEM (HRTEM) image (Fig. 1f). The distribution of elements in Bi_2_S_3_/Ti_3_C_2_ NHs was presented by the element mapping of energy dispersive X-ray spectroscopy (EDS) (Fig. 1g). All of these morphologically confirmed the synthesis of Bi_2_S_3_/Ti_3_C_2_ NHs. The X-ray diffraction (XRD) patterns demonstrated that the typical diffraction peaks of Bi_2_S_3_/Ti_3_C_2_ NHs could be indexed to the orthorhombic Bi_2_S_3_ phase (JCPDS No. 75-1306) and the hexagonal Ti_3_C_2_ phase (JCPDS No. 52–0875) (Fig. 1h and Additional file 1: Fig. S1), and the characteristic peaks of Bi_2_S_3_ and Ti_3_C_2_ were visible in the X-ray photoelectron spectroscopy (XPS) spectra of Bi_2_S_3_/Ti_3_C_2_ NHs (Fig. 1i, j and Additional file 1: Fig. S2). These two findings further verified the successful synthesis of Bi_2_S_3_/Ti_3_C_2_ NHs. The ultraviolet-visible (UV-vis) spectrum indicated that the absorption intensity of Bi_2_S_3_/Ti_3_C_2_ NHs in the NIR region was significantly higher than that of Bi_2_S_3_ NPs (Fig. 1k), which would be conducive to the improvement of PDT efficiency. The bandgap of Bi_2_S_3_ was calculated to be about 1.43 eV consistent with the previous report by the formula: ($\alpha$*h*$\nu$)^n^ = m (*h*$\nu$ - Eg) (Additional file 1: Fig. S3) [23], where $\alpha$, *h*, $\nu$ and Eg are absorption coefficient, Planck constant, photon frequency and bandgap energy, respectively, and n, m are constants. It satisfied the energy requirement for an 808 nm laser to excite NMs with a bandgap $<$ 1.53 eV [24]. Photoluminescence (PL) effect resulting from the combination of electrons and holes generated by photoexcitation could be used to evaluate the e^−^−h^+^ separation ability of PSs. The PL spectrum presented that the fluorescence intensity of Bi_2_S_3_/Ti_3_C_2_ NHs was substantially reduced compared to Bi_2_S_3_ NPs (Fig. 1l), with the greatest attenuation in the preparation ratio of 3:2 (Additional file 1: Fig. S4), suggesting that the e^−^−h^+^ separation efficiency of Bi_2_S_3_/Ti_3_C_2_ NHs was notably enhanced and useful for the further promotion of PDT efficiency.

To improve the biocompatibility and achieve the tumor-targeting capability of Bi_2_S_3_/Ti_3_C_2_ NHs, oil-soluble Bi_2_S_3_/Ti_3_C_2_ NHs were converted to water-soluble NHs, which were then encapsulated with methoxyployethylene glycol amine (mPEG-NH_2_) by electrostatic adsorption and attached with TPP on the surface via an amide bond (-NH-C = O-). Compared with the Fourier transform infrared (FT-IR) spectrum of Bi_2_S_3_/Ti_3_C_2_, the vibration of C-O-C at 1102 cm^-2^ from mPEG-NH_2_ and the characteristic absorption peaks consistent with the molecular structure of TPP, including benzene skeleton vibration at 1479 cm^-2^ and benzene ring monosubstituted peak vibration that appeared simultaneously at 750 cm^-2^ and 692 cm^-2^, were visible in the FT-IR spectrum of Bi_2_S_3_/Ti_3_C_2_-TPP (Fig. 1m). This analysis proved that mPEG-NH_2_ and TPP had been successfully conjugated on Bi_2_S_3_/Ti_3_C_2_ NHs. Dynamic light scattering (DLS) analysis presented the change in hydration particle size during each step of the Bi_2_S_3_/Ti_3_C_2_-TPP synthesis process (Additional file 1: Fig. S5a). The zeta potential measurements could also be utilized to represent the functionalized process of Bi_2_S_3_/Ti_3_C_2_ NHs (Additional file 1: Fig. S5b). The zeta potential of Ti_3_C_2_ NSs changed from − 27.2 mV $\pm$ 0.6 mV to 3.8 mV $\pm$ 0.8 mV after loading Bi_2_S_3_ NPs. The next encapsulation with mPEG-NH_2_ and attachment with TPP made the zeta potential change to -11.7 mV $\pm$ 1.0 mV and − 8.0 mV $\pm$ 0.2 mV, respectively. These results confirmed the completion of Bi_2_S_3_/Ti_3_C_2_-TPP fabrication. Furthermore, DLS analysis showed that Bi_2_S_3_/Ti_3_C_2_-TPP had good stability in pure water or Dulbecco’s modified eagle medium (DMEM), which would be beneficial for its application in biomedical research (Additional file 1: Fig. S5c and Fig. S6). Finally, the UV-vis spectra further verified that TPP was properly bonded to Bi_2_S_3_/Ti_3_C_2_ NHs (Additional file 1: Fig. S7a), and the standard curve of TPP aqueous solution was obtained by measuring UV-vis absorption spectra at different concentrations (Additional file 1: Fig. S7b, c), which was performed to calculate that the connection rate of TPP on Bi_2_S_3_/Ti_3_C_2_ NHs was 42.35%.

### Photodynamic and photothermal performance of Bi_2_S_3_/Ti_3_C_2_

**Fig. 2:**
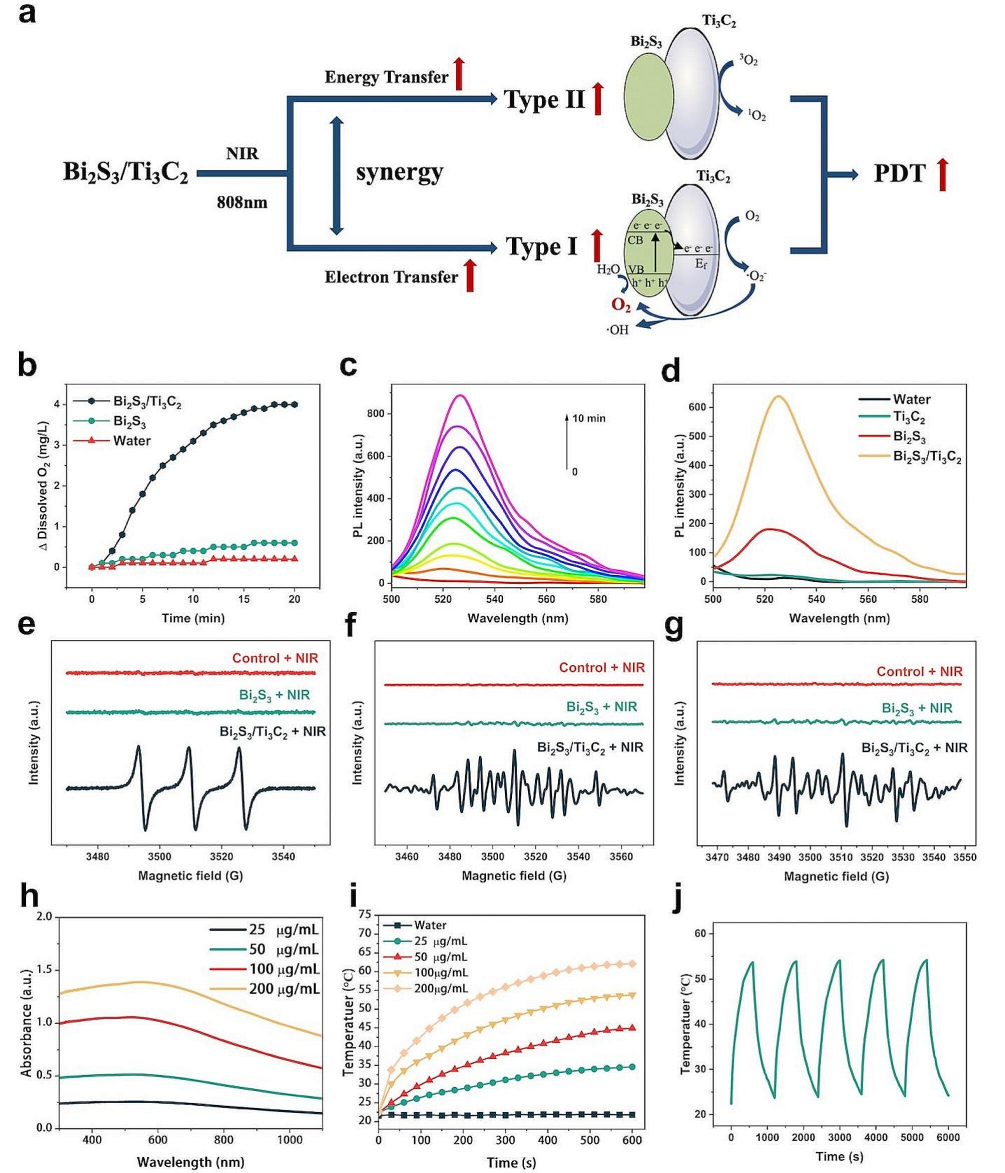
PDT mechanism of Bi_2_S_3_/Ti_3_C_2_ and its photodynamic and photothermal performance. **a** Illustration of Bi_2_S_3_/Ti_3_C_2_ as PSs for enhanced PDT through the synergetic effect of type I and type II PDT under NIR irradiation. **b** O_2_ production of Bi_2_S_3_/Ti_3_C_2_ and Bi_2_S_3_ aqueous solutions (100 µg mL^-1^) under 808 nm laser irradiation (1 W cm^-2^). **c** Time-dependent ROS generation by Bi_2_S_3_/Ti_3_C_2_ (100 µg mL^-1^) detected by DCFH-DA probe under 808 nm laser irradiation (1 W cm^-2^). **d** ROS generation of Ti_3_C_2_, Bi_2_S_3_, and Bi_2_S_3_/Ti_3_C_2_ (100 µg mL^-1^) under 808 nm laser irradiation (1 W cm^-2^) detected by DCFH-DA probe. ESR spectra of Bi_2_S_3_ and Bi_2_S_3_/Ti_3_C_2_ under 808 nm laser irradiation (1 W cm^-2^) for characterizing the generation of **e**^1^O_2_, **f** ·O_2_^−^, and **g** ·OH. **h** The UV–vis absorption spectra of Bi_2_S_3_/Ti_3_C_2_ with various concentrations. **i** Photothermal heating curves of Bi_2_S_3_/Ti_3_C_2_ with various concentrations under 808 nm laser irradiation (1 W cm^-2^). **j** Photostability of Bi_2_S_3_/Ti_3_C_2_ (100 µg mL^-1^) under 808 nm laser irradiation (1 W cm^-2^)

Under NIR irradiation, Bi_2_S_3_/Ti_3_C_2_ NHs generated ROS through the synergistic function of type I and type II PDT mechanisms and produced O_2_ simultaneously to enhance their therapeutic effect, as shown in Fig. 2a. Due to the fact that the Fermi energy level (E_F_) of Ti_3_C_2_ was lower than that of closely connected Bi_2_S_3_ in the Bi_2_S_3_/Ti_3_C_2_ NHs system [18, 25], a Schottky junction with a built-in electric field (E-field) could be formed at the contact interface of Ti_3_C_2_ and Bi_2_S_3_. According to the Mott-Schottky plots, the CB potential of Bi_2_S_3_ was about − 0.15 V vs. NHE (Additional file 1: Fig. S8), whereas the E_F_ of the O-terminated Ti_3_C_2_ was previously reported to be 0.71 eV vs. NHE [18, 26]. Thus, under 808 nm laser irradiation, the electrons of Bi_2_S_3_ were excited from VB to CB, resulting in activated electrons and holes, and the electrons were accelerated to transfer to Ti_3_C_2_ with an excellent conductivity property under the formation of an E-field that could effectively prevent their backflow to Bi_2_S_3_ [27]. This electron transfer process enhanced the separation efficiency of photoinduced electrons and holes in Bi_2_S_3_, as well as endowed Bi_2_S_3_/Ti_3_C_2_ NHs with strong redox capacity. The activated electrons could react with O_2_ to generate ·O_2_^-^ (Additional file S1: Eq. 1), while the activated holes could react with H_2_O to produce O_2_ (Additional file S1: Eq. 2). However, ·O_2_^-^, as a primary ROS, could be further transformed into ·OH with a stronger killing effect (Additional file S1: Eqs. 3, 4). The above ROS were generated through the electron transfer pathway of the type I PDT mechanism, and the O_2_ produced during the process could be converted into another cytotoxic ROS, ^1^O_2_, through the energy transfer pathway of the type II PDT mechanism. Therefore, under 808 nm laser irradiation, Bi_2_S_3_/Ti_3_C_2_ NHs could fulfill the synergistic effect of type I and type II PDT with or without abundant O_2_ to enhance PDT efficacy.

To emphasize the superiority of Bi_2_S_3_/Ti_3_C_2_ NHs, their abilities to generate O_2_ and ROS under NIR irradiation were explored. It was shown that Bi_2_S_3_/Ti_3_C_2_ NHs produced more O_2_ than Bi_2_S_3_ NPs under 808 nm laser irradiation (1 W cm^-2^) for 20 min by measuring the O_2_ content in sample solutions with a dissolved oxygen meter (Fig. 2b). The production of O_2_ could not only facilitate type II PDT, but also alleviate TME hypoxia. Whereafter, using a ROS assay kit in which non-fluorescent 2, 7-dichloro-dihydrofluorescein diacetate (DCFH-DA) could be oxidized by ROS to form fluorescent 2, 7-dichlorofluorescein diacetate (DCF), we found that the DCF fluorescence intensity in Bi_2_S_3_/Ti_3_C_2_ aqueous solution elevated progressively within 10 min under 808 nm laser irradiation (1 W cm^-2^), indicating the continuous generation of ROS (Fig. 2c). In addition, the DCF fluorescence intensity in Bi_2_S_3_/Ti_3_C_2_ aqueous solution was significantly stronger than that in both Bi_2_S_3_ NPs and Ti_3_C_2_ NSs aqueous solutions under 808 nm laser irradiation (1 W cm^-2^) (Fig. 2d), which verified that the photocatalytic efficiency of Bi_2_S_3_/Ti_3_C_2_ NHs was notably improved over the other two, since the formation of the Schottky junction with the existence of an E-field via the close integration of Bi_2_S_3_ and Ti_3_C_2_ could not only increase the rate of electron transfer but also prevent the combination of electrons and holes, thereby elevating the e^−^−h^+^ separation greatly [28]. Subsequently, the species of generated ROS were identified by electron spin-resonance (ESR) spectroscopy. In accordance with the detection results, the signal intensity of ^1^O_2_ in Bi_2_S_3_/Ti_3_C_2_ NHs aqueous solution was much higher than in Bi_2_S_3_ NPs aqueous solution under 808 nm laser irradiation (1 W cm^-2^, 10 min) (Fig. 2e), because Bi_2_S_3_/Ti_3_C_2_ NHs had the ability to produce O_2_ under NIR irradiation, which could increase the amount of ^1^O_2_ generated by energy transfer. Similarly, Bi_2_S_3_/Ti_3_C_2_ NHs aqueous solution exhibited strong ·O_2_^-^ (Fig. 2f) and ·OH (Fig. 2g) signals under the same irradiation conditions, whereas almost no signals were observed in Bi_2_S_3_ NPs aqueous solution. These findings further confirmed that Bi_2_S_3_/Ti_3_C_2_ NHs could raise the production of ^1^O_2_ through the O_2_-dependent type II PDT mechanism and generate ·O_2_^-^ and ·OH through the non-O_2_-dependent type I PDT mechanism.

Bi_2_S_3_/Ti_3_C_2_ NHs had strong absorption in the NIR region, and their extinction coefficient was obviously higher than that of Bi_2_S_3_ NPs and Ti_3_C_2_ NSs (Fig. 2h and Additional file 1: Fig. S9), indicating that Bi_2_S_3_/Ti_3_C_2_ NHs could exhibit more favorable photothermal performance, which was investigated under NIR irradiation. It was revealed that the Bi_2_S_3_/Ti_3_C_2_ aqueous solution manifested a concentration-dependent temperature rise curve by irradiating different concentrations of sample solutions with an 808 nm laser (1 W cm^-2^, 10 min) (Fig. 2i). Among them, the temperature of Bi_2_S_3_/Ti_3_C_2_ aqueous solution with a concentration of 200 $\mu$g mL^-1^ increased to 62.1 $^\circ C$ after NIR irradiation, while the temperature of pure water was only 21.9 $^\circ C$ under the same irradiation condition, demonstrating that Bi_2_S_3_/Ti_3_C_2_ aqueous solution had a remarkable photothermal effect. Based on the maximum steady-state temperature and the time constant for heat transfer acquired from the cooling curve, the PTCE of Bi_2_S_3_/Ti_3_C_2_ NHs was calculated to be 42.13% higher than that of Bi_2_S_3_ NPs (33.37%) and Ti_3_C_2_ NSs (29.89%) (Additional file 1: Fig. S10), which was also superior to those representative NMs with good photothermal effect in the NIR region, such as Au nanorods (21%) [29], MoS_2_ NSs (24.37%) [30], Cu_9_S_5_ nanocrystals (25.7%) [31], and Au nanovesicles (37%) [32]. Moreover, the temperature change of Bi_2_S_3_/Ti_3_C_2_ NHs aqueous solution was recorded under 808 nm laser irradiation (1 W cm^-2^) for 10 min (laser on) followed by natural cooling to room temperature (laser off), which was repeated for five cycles (Fig. 2j). The obtained profiles were generally consistent, suggesting that Bi_2_S_3_/Ti_3_C_2_ NHs had satisfactory photothermal stability and the potential to be employed for durable photothermal treatment of tumors. All of the above results revealed that Bi_2_S_3_/Ti_3_C_2_ NHs had excellent photothermal performance.

### Selection of a hypoxic tumor model

**Fig. 3:**
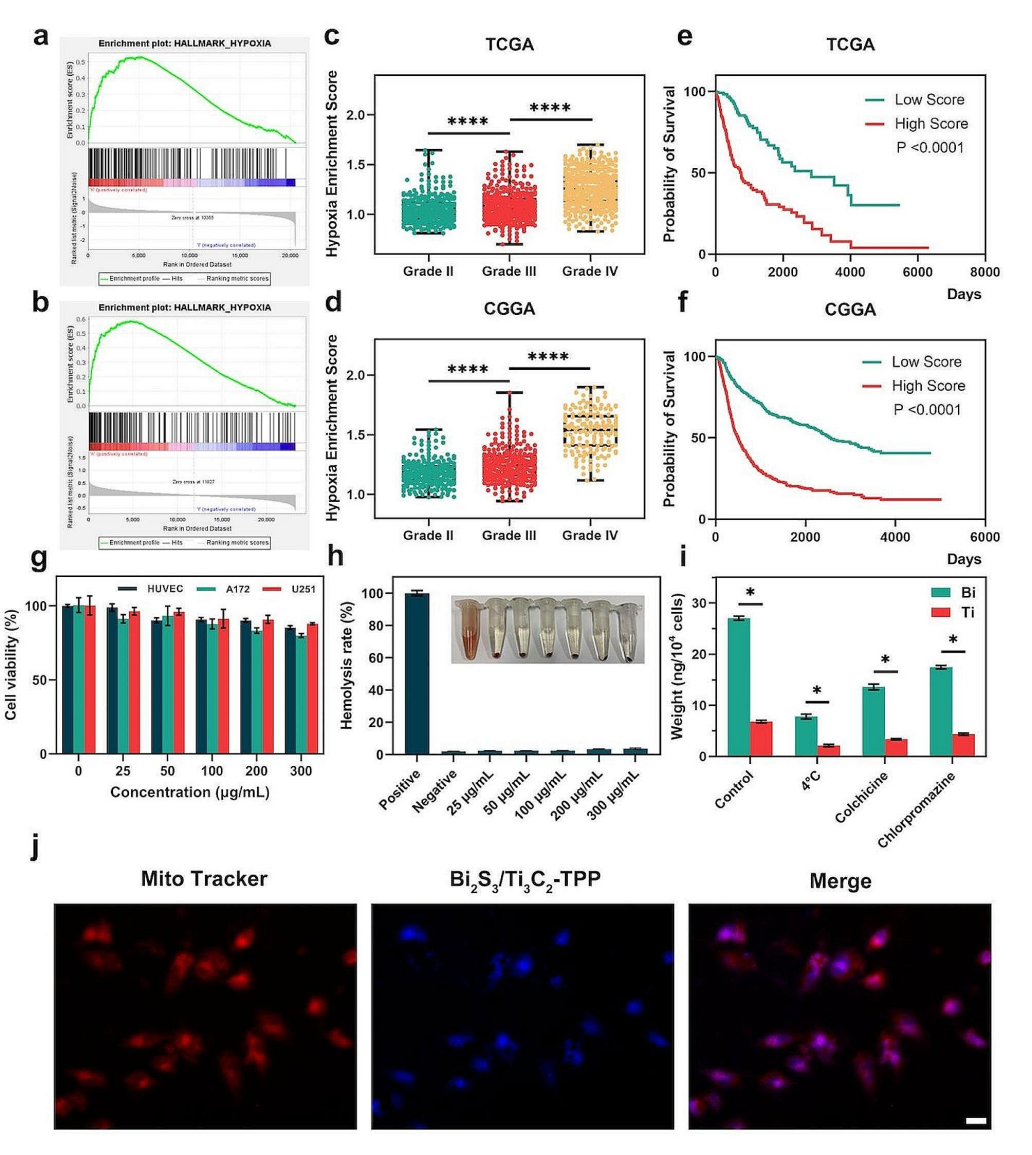
Hypoxic tumor model selection, biocompatibility, and uptake of Bi_2_S_3_/Ti_3_C_2_-TPP. The GSEA of hypoxia-related genes performed in **a** TCGA and **b** CGGA. The enrichment of hypoxia-related gene scores in gliomas of different grades in **c** TCGA and **d** CGGA; ****P < 0.0001. The relationship between hypoxia-related gene scores and patient survival time in **e** TCGA and **f** CGGA. **g** Cell viabilities of U251, A172, and HUVEC cells incubated with Bi_2_S_3_/Ti_3_C_2_-TPP at various concentrations. **h** Hemolysis quantification of erythrocytes at various concentrations of Bi_2_S_3_/Ti_3_C_2_-TPP. **i** The contents of Bi and Ti in U251 cells incubated with Bi_2_S_3_/Ti_3_C_2_-TPP measured by ICP-MS; *P < 0.05. **j** The image of Mito-Tracker Red stained U251 cells treated with Bi_2_S_3_/Ti_3_C_2_-TPP (100 µg mL^-1^, scale bar = 20 μm)

It was demonstrated that hypoxic-related genes had considerable functional enrichment in glioblastoma by enrichment analysis of the hypoxic-related gene set, HALLMARK_HYPOXIA.v2023.1.Hs.gmt, in The Cancer Genome Atlas (TCGA, https://portal.gdc.cancer.gov/) and Chinese Glioma Genome Atlas (CGGA, http://www.cgga.org.cn/) databases (Fig. 3a, b). Then, in order to better understand the enrichment of the hypoxic-related gene set in each glioma patient sample, we performed hypoxic-related functional scores on the samples in the TCGA and CGGA by single-sample gene set enrichment analysis (ssGSEA), which was further analyzed in conjunction with the clinical data. The results (Fig. 3c, d) revealed that hypoxia enrichment scores on the samples increased with tumor grade progression in the two databases. Among them, glioblastoma, the most aggressive glioma, had the highest hypoxia enrichment score. Finally, the joint analysis of the relationship between hypoxia enrichment score and prognosis time of glioma patients found that an increase in hypoxia enrichment score was negatively correlated with patient survival time (Fig. 3e, f). These findings indicated that hypoxia had become an essential element in the malignant progression of glioma. Thus, changing the hypoxic status in the TME of glioma might be an important means of delaying tumor progression, which is why glioma was chosen for the following biological experiments.

### Cytotoxicity and cellular uptake of Bi_2_S_3_/Ti_3_C_2_-TPP

The cytotoxicity of Bi_2_S_3_/Ti_3_C_2_-TPP was assessed by 3-(4,5-dimethyl-2-thiazolyl)-2,5-diphenyl-2 H-tetrazolium bromide (MTT) assays. The results showed that the cell viabilities of human glioma (U251) cells, human glioblastoma (A172) cells, and human umbilical vein endothelial (HUVEC) cells remained around 90%, even if the concentration of Bi_2_S_3_/Ti_3_C_2_-TPP was as high as 300 $\mu$g mL^-1^ (Fig. 3g), indicating that the toxicity of Bi_2_S_3_/Ti_3_C_2_-TPP to the above cells was negligible. In addition, for future biomedical research, the compatibility of Bi_2_S_3_/Ti_3_C_2_-TPP on mouse red blood cells was evaluated by hemolysis assays, and it was found that when the concentration of Bi_2_S_3_/Ti_3_C_2_-TPP was increased to 300 $\mu$g mL^-1^, the hemolysis rate was still less than 4% (Fig. 3h), implying that Bi_2_S_3_/Ti_3_C_2_-TPP had credible hemocompatibility.

As a prerequisite for tumor phototherapy, effective cellular uptake is considered necessary to achieve a good therapeutic effect. To systematically investigate the pathways and conditions of the cellular internalization of Bi_2_S_3_/Ti_3_C_2_-TPP, the content of two metallic elements, Bi and Ti, in tumor cells was determined by inductively coupled plasma-mass spectroscopy (ICP-MS) tests. As previously reported, low temperature could decrease adenosine triphosphate (ATP) synthesis and impede energy-dependent pathways [33]. As shown in Fig. 3i, when U251 cells were co-incubated with Bi_2_S_3_/Ti_3_C_2_-TPP at 4 $^\circ C$, the content of Bi or Ti in the cells detected by ICP-MS tests was only about 30% of that under the incubation condition of 37 $^\circ C$, illustrating that Bi_2_S_3_/Ti_3_C_2_-TPP endocytosis was primarily dependent on the energy-dependent pathways. Whereafter, colchicine and chlorpromazine (CPZ) were employed as macro-pinocytosis and clathrin-mediated endocytosis inhibitors to further explore the energy-dependent endocytosis mechanism of Bi_2_S_3_/Ti_3_C_2_-TPP. The results indicated that, compared to the control group, colchicine- and CPZ-treated cells inhibited Bi_2_S_3_/Ti_3_C_2_-TPP uptake by 49.9% and 35.8% (Additional file 1: Fig. S11a), respectively, more definitely suggesting that the endocytosis pathway of Bi_2_S_3_/Ti_3_C_2_-TPP was closely related to macro-pinocytosis and clathrin-mediated endocytosis. Moreover, after the incubation of U251 cells with Bi_2_S_3_/Ti_3_C_2_ or Bi_2_S_3_/Ti_3_C_2_-TPP for 4 h, the uptake of the two NMs in tumor cells showed a dose-dependent trend, with Bi_2_S_3_/Ti_3_C_2_-TPP being more significantly taken up than Bi_2_S_3_/Ti_3_C_2_ (Additional file 1: Fig. S11b, c). Thus, the targeting effect of TPP on tumor cells was confirmed. The tumor mitochondrial targeting performance of Bi_2_S_3_/Ti_3_C_2_-TPP could also be examined by staining U251 cells with Mito-Tracker Red. It was clearly observed by an inverted fluorescence microscope that the inherent blue fluorescence of Bi_2_S_3_/Ti_3_C_2_-TPP substantially overlapped with the red fluorescence of mitochondria (Fig. 3j), demonstrating that Bi_2_S_3_/Ti_3_C_2_-TPP was principally localized in the mitochondria and had excellent mitochondrial targeting performance. Meanwhile, because of TPP’s lipophilic cation characteristics, Bi_2_S_3_/Ti_3_C_2_-TPP might also diffuse to other subcellular organelles, such as the endoplasmic reticulum and lysosomes [34, 35].

### In vitro antitumor performance of Bi_2_S_3_/Ti_3_C_2_-TPP

**Fig. 4:**
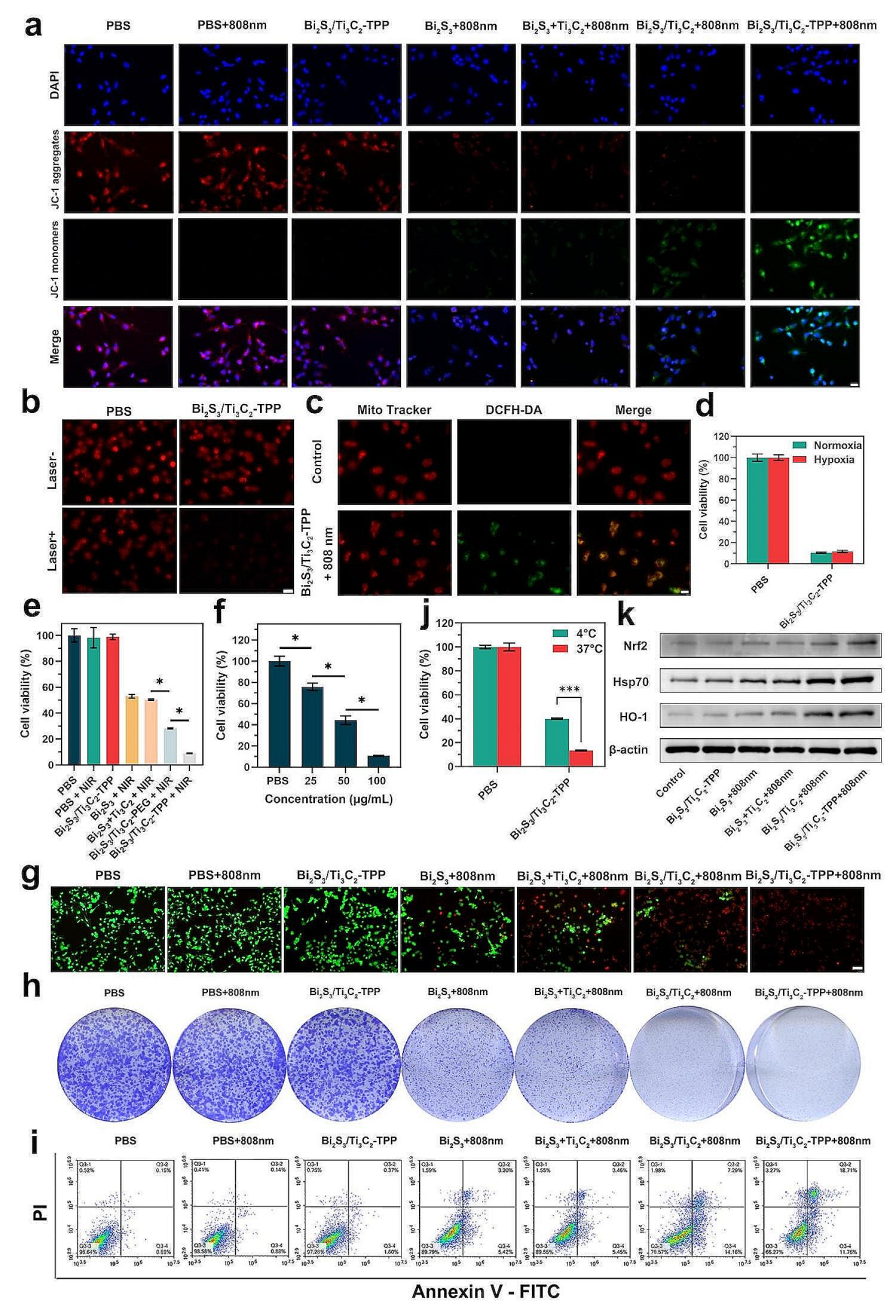
In vitro antitumor performance evaluation of Bi_2_S_3_/Ti_3_C_2_-TPP. **a** The images of JC-1 stained U251 cells after different treatments (scale bar = 20 μm). **b** Hypoxia level of PBS (pH = 7.4, 10 mM) and Bi_2_S_3_/Ti_3_C_2_-TPP (100 µg mL^-1^) treated U251 cells (scale bar = 50 μm). **c** ROS generation in U251 cells under 808 nm laser irradiation (1 W cm^-2^, 10 min, scale bar = 20 μm). **d** U251 cells treated with PBS (pH = 7.4, 10 mM) or Bi_2_S_3_/Ti_3_C_2_-TPP (100 µg mL^-1^) under 808 nm laser irradiation (1 W cm^-2^, 10 min) in N_2_ or air atmospheres. **e** MTT and **g** Calcein AM/PI two-staining analysis of U251 cells following various treatments (scale bar = 100 μm); *P < 0.05. **f** The viabilities of U251 cells treated with Bi_2_S_3_/Ti_3_C_2_-TPP at various concentrations under 808 nm laser irradiation (1 W cm^-2^, 10 min); *P < 0.05. **h** Cell clone formation of U251 cells after different treatments. **i** Flow cytometric analysis for apoptotic U251 cells by Annexin-V-FITC/PI assay. **j** U251 cells treated with PBS (pH = 7.4, 10 mM) or Bi_2_S_3_/Ti_3_C_2_-TPP (100 µg mL^-1^) under 808 nm laser irradiation (1 W cm^-2^, 10 min) at 37 $^\circ C$ and 4 $^\circ C$; ***P < 0.001. **k** Cellular Nrf2, Hsp70, and HO-1 expressions of U251 cells following various treatments

Mitochondrial depolarization could lead to the early apoptosis of cells [36]. JC-1 staining was used to monitor changes in mitochondrial membrane potential (MMP) to identify whether mitochondrial damage occurred following different experimental treatments. At normal MMP, JC-1 aggregated in the mitochondrial matrix, emitting red fluorescence, whereas at decreased MMP, it manifested a monomer state, emitting green fluorescence. As shown in Fig. 4a, U251 cells in the phosphate buffered saline (PBS, pH = 7.4, 10 mM), PBS with NIR irradiation (PBS + 808 nm, 1 W cm^-2^, 10 min), and Bi_2_S_3_/Ti_3_C_2_-TPP groups mainly exhibited strong red fluorescence. Green fluorescence was found in the Bi_2_S_3_, Bi_2_S_3_ + Ti_3_C_2_, and Bi_2_S_3_/Ti_3_C_2_ with NIR irradiation (Bi_2_S_3_ + 808 nm, Bi_2_S_3_ + Ti_3_C_2_ + 808 nm, and Bi_2_S_3_/Ti_3_C_2_ + 808 nm) groups, with the intensity of green fluorescence being stronger in the Bi_2_S_3_/Ti_3_C_2_ + 808 nm group than in the first two groups. Furthermore, the Bi_2_S_3_/Ti_3_C_2_-TPP with NIR irradiation (Bi_2_S_3_/Ti_3_C_2_-TPP + 808 nm) group remarkably showed the strongest green fluorescence. The fluoresceine isothiocyanate (FITC) channel of flow cytometry was performed to quantify the green fluorescence emitted by JC-1 monomer at decreased MMP in each group, and the results were consistent with the fluorescence images displayed above (Additional file 1: Fig. S12a). These studies illustrated that Bi_2_S_3_/Ti_3_C_2_-TPP could effectively accumulate in mitochondria and induce severe mitochondrial dysfunction under NIR irradiation.

Because adequate O_2_ supply could alleviate hypoxia in the TME and reduce resistance to the PDT effect, the O_2_-supplied capacity of Bi_2_S_3_/Ti_3_C_2_-TPP in tumor cells during PDT was investigated. Image-iT™, a red hypoxia probe, responded to decreased O_2_ levels in living cells by increasing its red fluorescence signal intensity in real time, which was utilized to detect intracellular O_2_ level. U251 cells in PBS with or without an 808 nm laser (1 W cm^-2^, 10 min) irradiation exhibited similar red fluorescence intensity. However, cells treated by Bi_2_S_3_/Ti_3_C_2_-TPP with NIR irradiation exhibited notably lower red fluorescence intensity than those without NIR irradiation (Fig. 4b), demonstrating that Bi_2_S_3_/Ti_3_C_2_-TPP had an excellent ability to split endogenous water and could produce sufficient O_2_ under NIR irradiation. The intracellular O_2_ supply of Bi_2_S_3_/Ti_3_C_2_-TPP with NIR irradiation could further induce the generation of enough lethal ROS in tumor cells (Fig. 4c), so that it was able to achieve a favorable antitumor effect under hypoxia comparable to that under normoxia (Fig. 4d). Flow cytometry was conducted to quantify the intracellular ROS levels, employing DCF fluorescence. Compared to the PBS group, the changes in DCF fluorescence intensity detected in the PBS + 808 nm and Bi_2_S_3_/Ti_3_C_2_-TPP groups were negligible. The groups of Bi_2_S_3_ + 808 nm, Bi_2_S_3_ + Ti_3_C_2_ + 808 nm, and Bi_2_S_3_/Ti_3_C_2_ + 808 nm could elicit DCF fluorescence enhancement, while the first two groups only displayed modest enhancement. Nevertheless, the Bi_2_S_3_/Ti_3_C_2_-TPP + 808 nm group considerably improved the DCF fluorescence intensity (Additional file 1: Fig. S12b), confirming the finding that Bi_2_S_3_/Ti_3_C_2_-TPP could generate abundant ROS by offering O_2_ under NIR irradiation to cause more serious oxidative injury to tumor cells.

The antitumor properties of Bi_2_S_3_/Ti_3_C_2_-TPP under NIR irradiation in vitro were thoroughly researched. According to MTT assays (Fig. 4e), the antitumor effects of the PBS, PBS + 808 nm, and Bi_2_S_3_/Ti_3_C_2_-TPP groups were marginal. The tumor cell viabilities of the Bi_2_S_3_ + 808 nm, Bi_2_S_3_ + Ti_3_C_2_ + 808 nm, and Bi_2_S_3_/Ti_3_C_2_ + 808 nm groups were 52.9%, 50.5%, and 28.2%, respectively. Moreover, the Bi_2_S_3_/Ti_3_C_2_-TPP + 808 nm group showed the best antitumor effect, with tumor cell viability reduced to 8.9%. The tumor cell viabilities of different concentrations of Bi_2_S_3_/Ti_3_C_2_-TPP under 808 nm laser (1 W cm^-2^, 10 min) irradiation were also examined (Fig. 4f), suggesting that 100 $\mu$g mL^-1^ was a proper concentration for antitumor therapy. Live/dead cell staining analysis further intuitively revealed that Bi_2_S_3_/Ti_3_C_2_-TPP under NIR irradiation could lead to more significant tumor cell death, which was compatible with the MTT assay results (Fig. 4g). Besides, the clone formation assay could reflect cell proliferation ability, which was carried out to discover that the Bi_2_S_3_/Ti_3_C_2_-TPP + 808 nm group possessed a lower cloning density and a substantially smaller number of cloned clusters than the control group (Fig. 4h), implying that this cell-treated condition had a strong inhibitory effect on tumor cell clone formation. Apoptosis was also found to be an essential marker of oxidative stress injury. Thus, flow cytometry was used to illustrate that there were almost no apoptotic and necrotic tumor cells in the PBS, PBS + 808 nm, and Bi_2_S_3_/Ti_3_C_2_-TPP groups (Fig. 4i). The proportions of apoptosis and necrosis triggered by Bi_2_S_3_ + 808 nm, Bi_2_S_3_ + Ti_3_C_2_ + 808 nm, and Bi_2_S_3_/Ti_3_C_2_ + 808 nm groups were 10.21%, 10.45%, and 23.43%, respectively, with the highest being 33.73% in the Bi_2_S_3_/Ti_3_C_2_-TPP + 808 nm group. Likewise, these outcomes confirmed that Bi_2_S_3_/Ti_3_C_2_-TPP under NIR irradiation could inhibit tumor cell proliferation by promoting their apoptosis, showing an outstanding in vitro antitumor effect. To determine the importance of ROS in the phototherapeutic effect of Bi_2_S_3_/Ti_3_C_2_-TPP, the contribution of PDT to total tumor cell death was assessed by performing MTT assays at 4 $^\circ C$ to restrain the PTT effect. As shown in Fig. 4j, the injury of Bi_2_S_3_/Ti_3_C_2_-TPP under NIR irradiation to tumor cells was weakened at 4 $^\circ C$ compared to 37 $^\circ C$, and the percentage of tumor cell death induced by PDT accounted for approximately 69.2%, emphasizing the leading role of Bi_2_S_3_/Ti_3_C_2_-TPP photodynamic performance in the antitumor process. All of these findings validated that Bi_2_S_3_/Ti_3_C_2_-TPP had fantastic in vitro antitumor capability, which was achieved through the synergistic and efficient therapeutic effect of multimode PDT combined with PTT.

Aiming to clarify the mechanism by which Bi_2_S_3_/Ti_3_C_2_-TPP caused tumor cell injury, western blot analysis was conducted on U251 cells treated under different experimental conditions to explore the relevant protein expressions. Nuclear factor erythroid 2-related factor 2 (Nrf2) is a basic leucine zipper protein that regulates the expression of antioxidant proteins like heme oxygenase-1 (HO-1), which protect organisms from oxidative damage via an antioxidant reaction. These two proteins’ expression could reflect the cellular damage induced by PDT. The expression of heat-responsive heat shock protein 70 (Hsp70) was frequently utilized to evaluate the damage caused by PTT-triggered intracellular hyperthermia. As shown in Fig. 4k and Additional file 1: Fig. S13, the expressions of Nrf2, HO-1, and Hsp70 in the group of Bi_2_S_3_/Ti_3_C_2_-TPP without an 808 nm laser (1 W cm^-2^, 10 min) irradiation were not significantly different from those in the control group. The expressions of these three proteins in the Bi_2_S_3_ + 808 nm, Bi_2_S_3_ + Ti_3_C_2_ + 808 nm, and Bi_2_S_3_/Ti_3_C_2_ + 808 nm groups were all elevated compared to the control group, and the expressions in the Bi_2_S_3_/Ti_3_C_2_ + 808 nm group were higher than those in the first two groups. Additionally, the expressions of Nrf2, HO-1, and Hsp70 in the Bi_2_S_3_/Ti_3_C_2_-TPP + 808 nm group were the highest. The results indicated that Bi_2_S_3_/Ti_3_C_2_-TPP could more effectively stimulate the oxidative stress pathway and had a superior thermal response under NIR irradiation.

### Biosafety and biodistribution of Bi_2_S_3_/Ti_3_C_2_-TPP

**Fig. 5:**
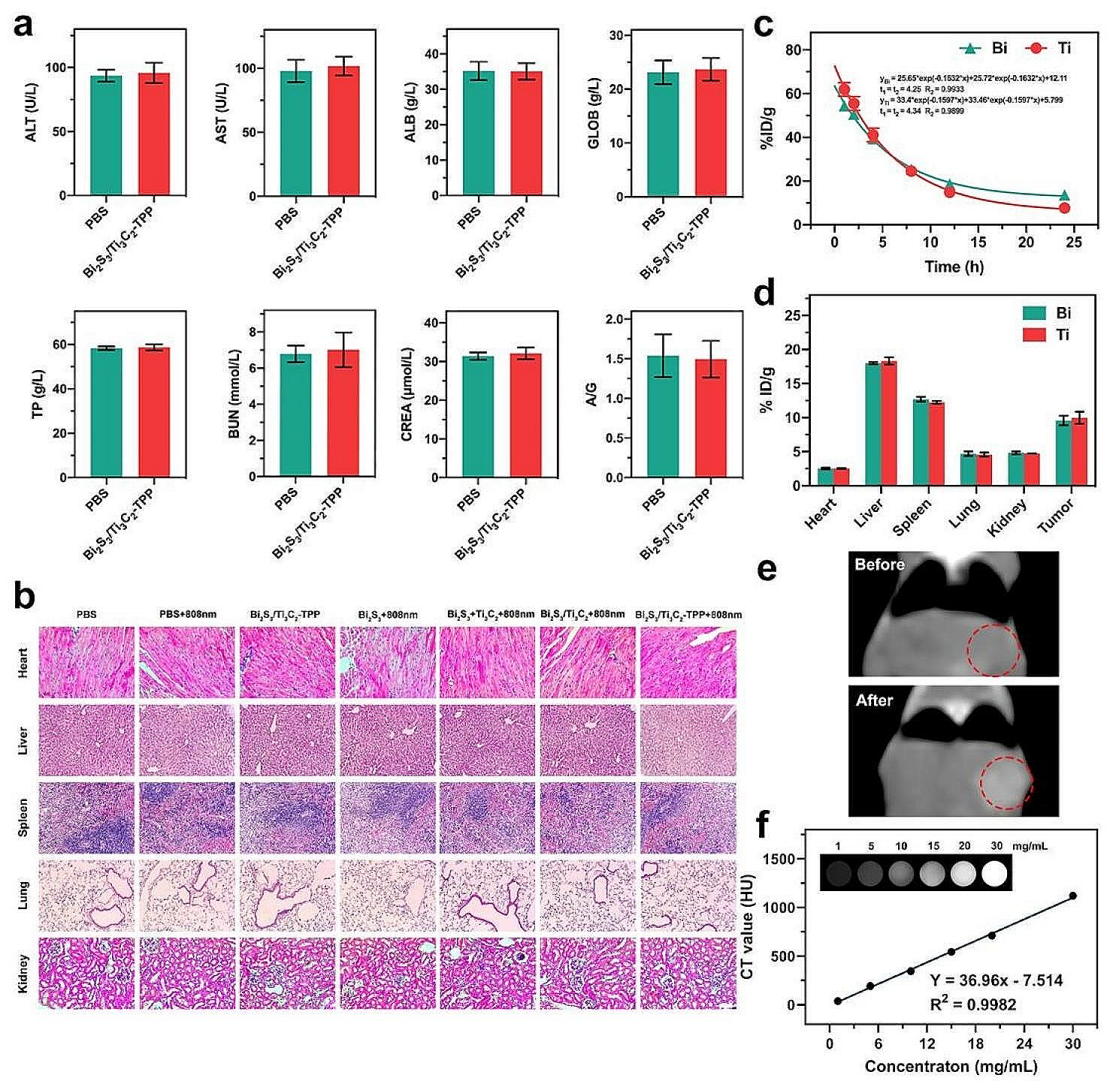
Biosafety and biodistribution evaluation of Bi_2_S_3_/Ti_3_C_2_-TPP. **a** Serum biochemistry results involving ALB, ALT, AST, TP, GLOB, BUN, CREA, and A/G ratio obtained from mice injected with PBS (pH = 7.4, 10 mM) or Bi_2_S_3_/Ti_3_C_2_-TPP (20 mg kg^-1^) at 24 h. **b** The pictures of H&E dyed primary apparatus of mice involving heart, liver, spleen, lung, and kidney following various treatments (scale bar = 40 μm). **c** Blood circulation and **d** biodistribution of Bi_2_S_3_/Ti_3_C_2_-TPP (20 mg kg^-1^) after intravenous injection into U251 tumor-bearing mice by measuring Bi and Ti concentrations with ICP-MS. **e** In vivo CT pictures of U251 tumor-bearing mice before and after injecting with Bi_2_S_3_/Ti_3_C_2_-TPP (20 mg kg^-1^) intravenously. **f** In vitro CT images and CT values of Bi_2_S_3_/Ti_3_C_2_-TPP at various concentrations

Prior to exploring the in vivo antitumor properties of Bi_2_S_3_/Ti_3_C_2_-TPP, we investigated its potential toxicity to better understand the biosafety of Bi_2_S_3_/Ti_3_C_2_-TPP. Routine blood biochemical indices of mice were detected, including alanine aminotransferase (ALT), alanine aminotransferase (AST), albumin (ALB), globulin (GLOB), total protein (TP), blood urea nitrogen (BUN), and creatinine (CREA), and the ratio of ALB to GLOB (A/G) was calculated. As shown in Fig. 5a, the mice treated with Bi_2_S_3_/Ti_3_C_2_-TPP were not abnormal compared to the control group, suggesting that Bi_2_S_3_/Ti_3_C_2_-TPP had no evident hepatic and renal cytotoxicity. Furthermore, the major organs (heart, liver, spleen, lung, and kidney) of each group after 2 weeks of experimental treatments were sliced, and hematoxylin and eosin (H&E) staining was performed. The results (Fig. 5b**)** demonstrated no substantial inflammation, damage, or chronic pathological toxicity, verifying the good biocompatibility of Bi_2_S_3_/Ti_3_C_2_-TPP in vivo. Both studies showed that Bi_2_S_3_/Ti_3_C_2_-TPP had favorable biosafety.

The blood circulation of Bi_2_S_3_/Ti_3_C_2_-TPP was consistent with a two-compartment model, and its half-life was calculated to be approximately 4.3 h (Fig. 5c**)**, allowing Bi_2_S_3_/Ti_3_C_2_-TPP to achieve efficient accumulation at the tumor site for treatment. Similarly, the contents of Bi and Ti elements in major organs and tumors of mice collected after caudal vein injection of Bi_2_S_3_/Ti_3_C_2_-TPP for 24 h were detected by ICP-MS tests (Fig. 5d**)**. High tumor accumulation of Bi_2_S_3_/Ti_3_C_2_-TPP (${\sim}$ 9.78% ID/g) was found due to the enhanced permeability and retention (EPR) effect, and the high levels of it in the major metabolic organ, liver and spleen, revealed that Bi_2_S_3_/Ti_3_C_2_-TPP could be cleared from the organism through these two organs. CT images of mice at 8 h post-injection further confirmed the significant accumulation of Bi_2_S_3_/Ti_3_C_2_-TPP in tumor tissues (Fig. 5e, f**)**, indicating that Bi_2_S_3_/Ti_3_C_2_-TPP could be employed as a CT imaging agent to benefit in tumor diagnosis and guided treatment, ensuring a satisfactory tumor therapeutic outcome.

### In vivo antitumor Performance of Bi_2_S_3_/Ti_3_C_2_-TPP

**Fig. 6:**
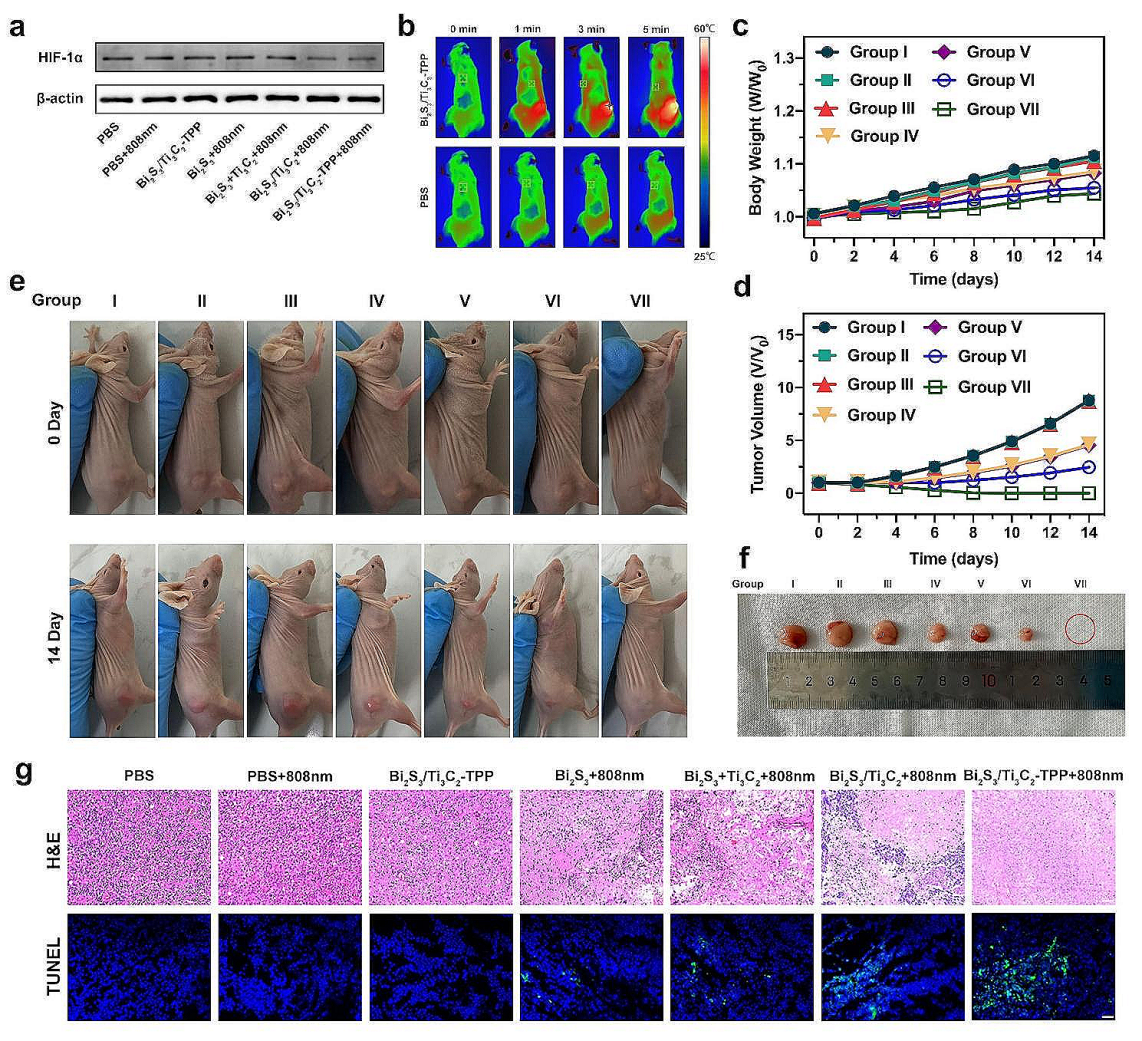
In vivo antitumor performance evaluation of Bi_2_S_3_/Ti_3_C_2_-TPP. **a** HIF-1α expressions of tumors after different treatments. **b** Infrared thermography of U251 tumor-bearing mice after being injected with PBS (pH = 7.4, 10 mM) or Bi_2_S_3_/Ti_3_C_2_-TPP (20 mg kg^-1^). Time-dependent relative **c** body mass and **d** tumor dimension change plots from various groups. **e** The photos of U251 tumor-bearing mice from various groups during two weeks. **f** The representative photo of tumors dissected at 14 days post-injection. **g** Representative images of H&E (scale bar = 100 μm) and TUNEL (scale bar = 20 μm) stained slices of tumor tissues after different treatments

Bi_2_S_3_/Ti_3_C_2_-TPP showed excellent synergistic effect of PDT and PTT in tumor cells in vitro, implying that Bi_2_S_3_/Ti_3_C_2_-TPP would have an outstanding tumor therapeutic effect in vivo. Balb/c U251 tumor-bearing nude mice were randomly divided into 7 treatment groups (*n* = 5) for different treatments to investigate the antitumor performance of Bi_2_S_3_/Ti_3_C_2_-TPP in vivo: Group I: caudal vein injection of PBS (pH = 7.4, 10 mM); Group II: PBS with an 808 nm laser irradiation (1 W cm^-2^, 10 min); Group III: Bi_2_S_3_/Ti_3_C_2_-TPP (20 mg kg^-1^); Group IV: Bi_2_S_3_ with an 808 nm laser irradiation; Group V: Bi_2_S_3_ + Ti_3_C_2_ with an 808 nm laser irradiation (dose ratio 3:2); Group VI: Bi_2_S_3_/Ti_3_C_2_ with an 808 nm laser irradiation; Group VII: Bi_2_S_3_/Ti_3_C_2_-TPP with an 808 nm laser irradiation. Firstly, the O_2_ production capacity of Bi_2_S_3_/Ti_3_C_2_-TPP in vivo under NIR irradiation was examined by detecting the expression of hypoxia-inducible factor-1α (HIF-1α) in tumor tissues. HIF-1α expression in the tumor tissues of Group VII was dramatically down-regulated, suggesting that the tumor hypoxic microenvironment was successfully alleviated (Fig. 6a and Additional file 1: Fig. S14). Subsequently, temperatures of the tumor sites in mice under NIR irradiation were measured with a portable thermal imager and recorded, aiming to explore the photothermal performance in vivo. As shown in Fig. 6b and Additional file 1: Fig. S15, the tumor-site temperature of mice treated with Bi_2_S_3_/Ti_3_C_2_-TPP increased from 31.2 $^\circ C$ to 51.9 $^\circ C$ after an 808 nm laser irradiation (1 W cm^-2^) for 5 min, demonstrating that Bi_2_S_3_/Ti_3_C_2_-TPP had strong photothermal conversion ability in vivo. Meanwhile, the body weights and tumor volumes of the mice were monitored every other day within two weeks after treatments, and photographs were taken to evaluate the therapeutic effect of each group. According to the results (Fig. 6c-f**)**, the control groups (Group I, II, and III) had no apparent inhibitory effect on tumor growth in mice. Groups IV, V, and VI were observed to have a therapeutic impact on mouse tumors, and the inhibition degree of tumor growth in Group VI was higher than in Group IV and V owing to the combination of multimodal PDT and PTT. Notably, the tumor growth in group VII was entirely inhibited, attributed to the tumor mitochondria-targeting property conferred by TPP, and no tumor recurrence was found over two weeks, which illustrated the exceptional phototherapy effect of Bi_2_S_3_/Ti_3_C_2_-TPP under NIR irradiation. Furthermore, there was no noteworthy change in mouse body weight amongst all experimental groups during two weeks, indicating that the systemic side effects of NMs were negligible. H&E and transferase-mediated deoxyuridine triphosphate nick end labeling (TUNEL) staining results (Fig. 6g**)** revealed that no inflammation or damage was observed in tumor cells in the control groups (Group I, II, and III). There was tiny focal or fragmentary tumor cell necrosis in Group IV, V, and VI, and the necrosis degree in Group VI was more severe than in Group IV and V, while Group VII had the largest proportion of tumor cell necrosis. These further confirmed that the in vivo anti-tumor performance of Bi_2_S_3_/Ti_3_C_2_-TPP was extraordinary under NIR irradiation.

## Conclusion

In summary, Bi_2_S_3_/Ti_3_C_2_-TPP was designed to produce O_2_ and ROS synchronously via type I and type II PDT mechanisms, combined with PTT under NIR irradiation at the same wavelength for synergistic phototherapy of hypoxic tumors. The formation of Bi_2_S_3_/Ti_3_C_2_ NHs not only enhanced the separation efficiency of photoinduced electrons and holes, but also endowed them with strong redox capacity. Actually, after the modification of TPP on Bi_2_S_3_/Ti_3_C_2_ NHs, Bi_2_S_3_/Ti_3_C_2_-TPP showed favorable biocompatibility and could be effectively internalized into tumor cells by targeting the mitochondria, which induced more severe tumor cell damage and elimination under NIR irradiation and exhibited excellent CT imaging capability. Therefore, Bi_2_S_3_/Ti_3_C_2_-TPP expands the phototherapeutic effect of Bi_2_S_3_-based nanoplatforms and holds a great prospect in hypoxic tumor theranostics.

### Electronic supplementary material

Below is the link to the electronic supplementary material.

Supplementary Material 1

## Data Availability

The data that support the findings of this study are available from the corresponding author upon reasonable request.

## References

[1] Li W, Zhang G, Liu L (2021). Near-Infrared Inorganic nanomaterials for Precise diagnosis and therapy. Front Bioeng Biotechnol.

[2] Wang K, Xue SS, Liu X, Pan W, Li N, Tang B (2021). Stimuli-activated molecular photothermal agents for cancer therapy. Chem Commun (Camb).

[3] Ji B, Wei M, Yang B (2022). Recent advances in nanomedicines for photodynamic therapy (PDT)-driven cancer immunotherapy. Theranostics.

[4] Yang Z, Luo Y, Yu H, Liang K, Wang M, Wang Q, Yin B, Chen H (2022). Reshaping the Tumor Immune Microenvironment based on a light-activated nanoplatform for efficient Cancer therapy. Adv Mater.

[5] Hu H, Liu X, Hong J, Ye N, Xiao C, Wang J, Li Z, Xu D (2022). Mesoporous polydopamine-based multifunctional nanoparticles for enhanced cancer phototherapy. J Colloid Interface Sci.

[6] de Melo-Diogo D, Lima-Sousa R, Alves CG, Correia IJ (2019). Graphene family nanomaterials for application in cancer combination photothermal therapy. Biomater Sci.

[7] Sun J, Li Y, Teng Y, Wang S, Guo J, Wang C (2020). NIR-controlled HSP90 inhibitor release from hollow mesoporous nanocarbon for synergistic tumor photothermal therapy guided by photoacoustic imaging. Nanoscale.

[8] Sheng Z, Hu D, Zheng M, Zhao P, Liu H, Gao D, Gong P, Gao G, Zhang P, Ma Y, Cai L (2014). Smart human serum albumin-indocyanine green nanoparticles generated by programmed assembly for dual-modal imaging-guided cancer synergistic phototherapy. ACS Nano.

[9] Li J, Dai S, Qin R, Shi C, Ming J, Zeng X, Wen X, Zhuang R, Chen X, Guo Z, Zhang X (2021). Ligand Engineering of Titanium-Oxo Nanoclusters for Cerenkov Radiation-Reinforced Photo/Chemodynamic Tumor Therapy. ACS Appl Mater Interfaces.

[10] Qiu M, Wang D, Huang H, Yin T, Bao W, Zhang B, Xie Z, Xie N, Wu Z, Ge C (2021). A Regioselectively oxidized 2D Bi/BiOx lateral Nano-Heterostructure for hypoxic photodynamic therapy. Adv Mater.

[11] Bejjanki NK, Zhong Y, Liu J, Li Q, Xu H, Shen H, Xie M (2021). Surface charge transition nano-theranostics based on ultra-small Fe(3)O(4) nanoparticles for enhanced photodynamic and photothermal therapy against nasopharyngeal carcinoma. Biochem Biophys Res Commun.

[12] Zhang Q, Li Y, Zhu S, Liu R, Zhu H (2022). AIPE-Active ir(III) complexes with tuneable photophysical properties and application in mitochondria-targeted dual-mode photodynamic therapy. Spectrochim Acta Mol Biomol Spectrosc.

[13] Zhao J, Zhang L, Qi Y, Liao K, Wang Z, Wen M, Zhou D (2021). NIR Laser Responsive nanoparticles for Ovarian Cancer targeted combination therapy with Dual-Modal Imaging Guidance. Int J Nanomed.

[14] Ouyang R, Cao P, Jia P, Wang H, Zong T, Dai C, Yuan J, Li Y, Sun D, Guo N (2021). Bistratal Au@Bi(2)S(3) nanobones for excellent NIR-triggered/multimodal imaging-guided synergistic therapy for liver cancer. Bioact Mater.

[15] Luo K, Zhao J, Jia C, Chen Y, Zhang Z, Zhang J, Huang M, Wang S (2020). Integration of Fe(3)O(4) with Bi(2)S(3) for Multi-modality Tumor Theranostics. ACS Appl Mater Interfaces.

[16] Wang S, Li X, Chen Y, Cai X, Yao H, Gao W, Zheng Y, An X, Shi J, Chen H (2015). A Facile One-Pot synthesis of a two-dimensional MoS2 /Bi2S3 Composite Theranostic Nanosystem for Multi-modality Tumor Imaging and Therapy. Adv Mater.

[17] Yao J, Zhu C, Peng T, Ma Q, Gao S (2021). Injectable and temperature-sensitive Titanium Carbide-Loaded Hydrogel System for Photothermal therapy of breast Cancer. Front Bioeng Biotechnol.

[18] Cao SWS, Tong BJ, Fu T, Yu JW (2018). 2D/2D heterojunction of ultrathin MXene/Bi2WO6 nanosheets for Improved Photocatalytic CO2 reduction. Adv Funct Mater.

[19] Liu G, Zou J, Tang Q, Yang X, Zhang Y, Zhang Q, Huang W, Chen P, Shao J, Dong X (2017). Surface modified Ti(3)C(2) MXene Nanosheets for Tumor Targeting Photothermal/Photodynamic/Chemo synergistic therapy. ACS Appl Mater Interfaces.

[20] Han X, Huang J, Lin H, Wang Z, Li P, Chen Y (2018). 2D ultrathin MXene-Based drug-delivery nanoplatform for synergistic photothermal ablation and chemotherapy of Cancer. Adv Healthc Mater.

[21] Cheng Y, Chang Y, Feng Y, Jian H, Tang Z, Zhang H (2018). Deep-level defect enhanced Photothermal performance of Bismuth Sulfide-Gold Heterojunction Nanorods for Photothermal Therapy of Cancer guided by computed Tomography Imaging. Angew Chem Int Ed Engl.

[22] Liang R, Li Y, Huo M, Lin H, Chen Y (2019). Triggering Sequential Catalytic Fenton reaction on 2D MXenes for hyperthermia-augmented synergistic Nanocatalytic Cancer Therapy. ACS Appl Mater Interfaces.

[23] Raoa M, Wub J, Asiric A, Anandan S (2021). Rice grain like Bi2S3 nanorods and its photocatalytic performance. Mater Sci Eng B.

[24] Wang W, Yang J, Zou Z, Zhang J, Li H, Wang Y (2018). An isolated deep-trap phosphor for optical data storage. Ceram Int.

[25] Paul S, Ghosh S, Barman D, De S (2017). Maximization of photocatalytic activity of Bi2S3/TiO2/Au ternary heterostructures by proper epitaxy formation and plasmonic sensitization. Appl Catal B-Environ.

[26] Chertopalov S, Mochalin V (2018). Environment Sensitive Photoresponse of spontaneously. ACS Nano.

[27] Yao Z, Huang Y, Zhu L, Obraztsov P, Du W, Zhang L, Xu X (2019). Interfacial THz Generation from Graphene/Si mixed-dimensional Van Der Waals Heterostructure. Nanoscale.

[28] Azadmanjiri J, Srivastava V, Kumar P, Wang J, Yu A (2018). Graphene-supported 2D transition metal oxide heterostructures. J Mater Chem A.

[29] Zeng J, Goldfeld D, Xia Y (2013). A plasmon-assisted optofluidic (PAOF) system for measuring the photothermal conversion efficiencies of gold nanostructures and controlling an electrical switch. Angew Chem Int Ed Engl.

[30] Yin W, Yan L, Yu J, Tian G, Zhou L, Zheng X, Zhang X, Yong Y, Li J, Gu Z, Zhao Y (2014). High-throughput synthesis of single-layer MoS2 nanosheets as a near-infrared photothermal-triggered drug delivery for effective cancer therapy. ACS Nano.

[31] Tian Q, Jiang F, Zou R, Liu Q, Chen Z, Zhu M, Yang S, Wang J, Wang J, Hu J (2011). Hydrophilic Cu9S5 nanocrystals: a photothermal agent with a 25.7% heat conversion efficiency for photothermal ablation of cancer cells in vivo. ACS Nano.

[32] Huang P, Lin J, Li W, Rong P, Wang Z, Wang S, Wang X, Sun X, Aronova M, Niu G (2013). Biodegradable gold nanovesicles with an ultrastrong plasmonic coupling effect for photoacoustic imaging and photothermal therapy. Angew Chem Int Ed Engl.

[33] Park S, Chun S, Kim D (2013). Cold exposure lowers energy expenditure at the cellular level. Cell Biol Int.

[34] Qin B, Yuan X, Jiang M, Yin H, Luo Z, Zhang J, Zhu C, Li X, Shi Y, Luo L (2020). Targeting DNA to the endoplasmic reticulum efficiently enhances gene delivery and therapy. Nanoscale.

[35] Banik SM, Pedram K, Wisnovsky S, Ahn G, Riley NM, Bertozzi CR (2020). Lysosome-targeting chimaeras for degradation of extracellular proteins. Nature.

[36] Roy S, Singh M, Rawat A, Kumar D, Kaithwas G (2020). Mitochondrial apoptosis and curtailment of hypoxia-inducible factor-1alpha/fatty acid synthase: a dual edge perspective of gamma linolenic acid in ER + mammary gland cancer. Cell Biochem Funct.

