# Supplementary material for: Bi2S3/Ti3C2-TPP nano-heterostructures induced by near-infrared for photodynamic therapy combined with photothermal therapy on hypoxic tumors
Source: J Nanobiotechnology. 2024 Mar 20;22:123. doi: 10.1186/s12951-024-02391-x (PMC10953153; doi:10.1186/s12951-024-02391-x)
Supplement: Supplementary file 1 — Supplementary Material 1 [file 12951_2024_2391_MOESM1_ESM.docx]

**Supporting Information**

Bi_2_S_3_/Ti_3_C_2_-TPP Nano-Heterostructures Induced by Near-Infrared for Photodynamic Therapy Combined with Photothermal Therapy on Hypoxic Tumors

Hanwen Jiang^1,2^, Jingxian Sun^3,5^, Fucong Liu^1^, Yuanjiao Zhao^1^, Xin Chen^3^, Changsong Dai^4^, and Zhaohui Wen^1^*.

^1^ Department of Neurology, Brain Ultrasound, The First Afﬁliated Hospital of Harbin Medical University, Harbin 150001, Heilongjiang Province, China

^2^ Department of Cardiology, Cardiac Ultrasound, The First Afﬁliated Hospital of Harbin Medical University, Harbin 150001, Heilongjiang Province, China

^3^ Key Colleges and Universities Laboratory of Neurosurgery in Heilongjiang Province, Harbin 150001, Heilongjiang Province, China

^4^ MIIT Key Laboratory of Critical Materials Technology for New Energy Conversion and Storage, School of Chemistry and Chemical Engineering, Harbin Institute of Technology, Harbin 150001 China

^5^ Department of Neurosurgery, the Affiliated Hospital of Qingdao University, Qingdao 266005, Shandong Province, China

Email: [wenzhaohui1968@163.com](mailto:wenzhaohui1968@163.com)

**A: Calculation of Photothermal Conversion Efficiency**

Following the Roper report [1], the total energy balance for the system is

$\sum_{i} m_{i}C_{p,i}\frac{dT}{dt}$ = $Q_{NMs}$ + $Q_{Dis}$ – $Q_{surr}$

in which $m_{i}$ and $C_{p,i}$ represent the mass and heat capacity of system components, respectively. T represents the solution temperature, $Q_{Dis}$ represents the basic energy input of the quartz cuvette cell containing pure water without samples, and $Q_{surr}$ represents the heat conducted away from the system surface by air. For the heat output:

$Q_{surr}$ = $hS(T{- T}_{surr})$

Where $h$ is the heat transfer coefficient, $S$ is the surface area of the container, and $T_{surr}$ is the ambient temperature of the surroundings. The system temperature will rise to a maximum when the heat input is equal to the heat output:

$Q_{NMs}$ + $Q_{Dis}$ = $Q_{surr - max}$ = $hS(T_{max}{- T}_{surr})$

In the above equation, $Q_{surr -max}$ is heat conduction away from the system surface by air when the sample cell reaches the equilibrium temperature, and $T_{max}$ is the maximum system temperature.

The photothermal conversion efficiency is calculated from the following equation:

$\eta$ *=* $\frac{hS{(T}_{max}- T_{surr}) - Q_{Dis}}{I(1- {10}^{{-A}_{808}})}$

Where $I$ is the laser power and $A_{808}$ is the absorbance of the samples at the wavelength of 808 nm.

In order to get the $hS$, a dimensionless driving force temperature, $\theta$ is introduced as follows:

$\theta$ = $\frac{T- T_{surr}}{T_{max}- T_{surr}}$

And a sample system time constant $\tau_{s}$,

$\tau_{s}$ = $\frac{\sum_{i} m_{i}C_{p,i}}{hS}$

Thus, $\frac{d\theta}{dt}$ = $\frac{1}{\tau_{s}}\frac{Q_{NMs} + Q_{Dis}}{hS(T_{max}{- T}_{surr})}$ – $\frac{\theta}{\tau_{s}}$, $\frac{d\theta}{dt}$ = – $\frac{\theta}{\tau_{s}}$, $t$ = –$\tau_{s}ln\theta$

So $hS$ could be calculated from the slope of cooling time *vs.* $ln\theta$.

**Reference:**

1. Roper DK, Ahn W, Hoepfner M. Microscale Heat Transfer Transduced by Surface Plasmon Resonant Gold Nanoparticles. J Phys Chem C Nanomater Interfaces 2007, 111:3636-3641.

**B: Reaction Equations**

**Equation 1**  O_2_ + e^-^ $\to$ ·O_2_^-^

**Equation 2** 2H_2_O + 4h^+^ $\to$ O_2_ + 4H^+^

**Equation 3** ·O_2_^-^ + e^-^ + 2H^+^ $\to$ H_2_O_2_

**Equation 4** ·O_2_^-^ + H_2_O_2_ + 2h^+^ $\to$ ·OH + OH^-^ + O_2_

**C: Supplementary Figures**

**
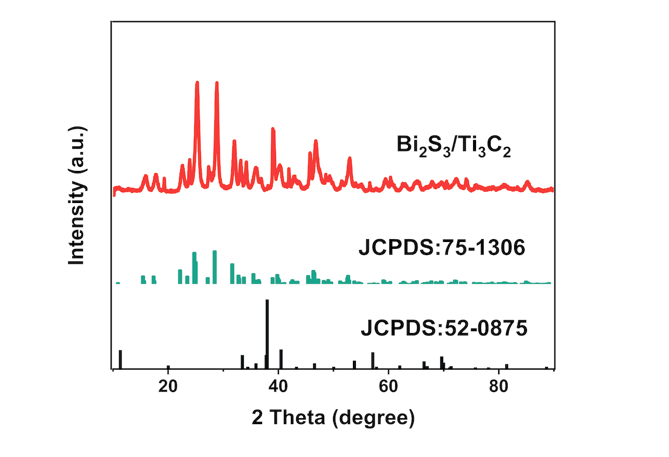
**

**Fig. S1** XRD patterns of Bi_2_S_3_ NPs, Ti_3_C_2_ NSs, and Bi_2_S_3_/Ti_3_C_2_ NHs.

**
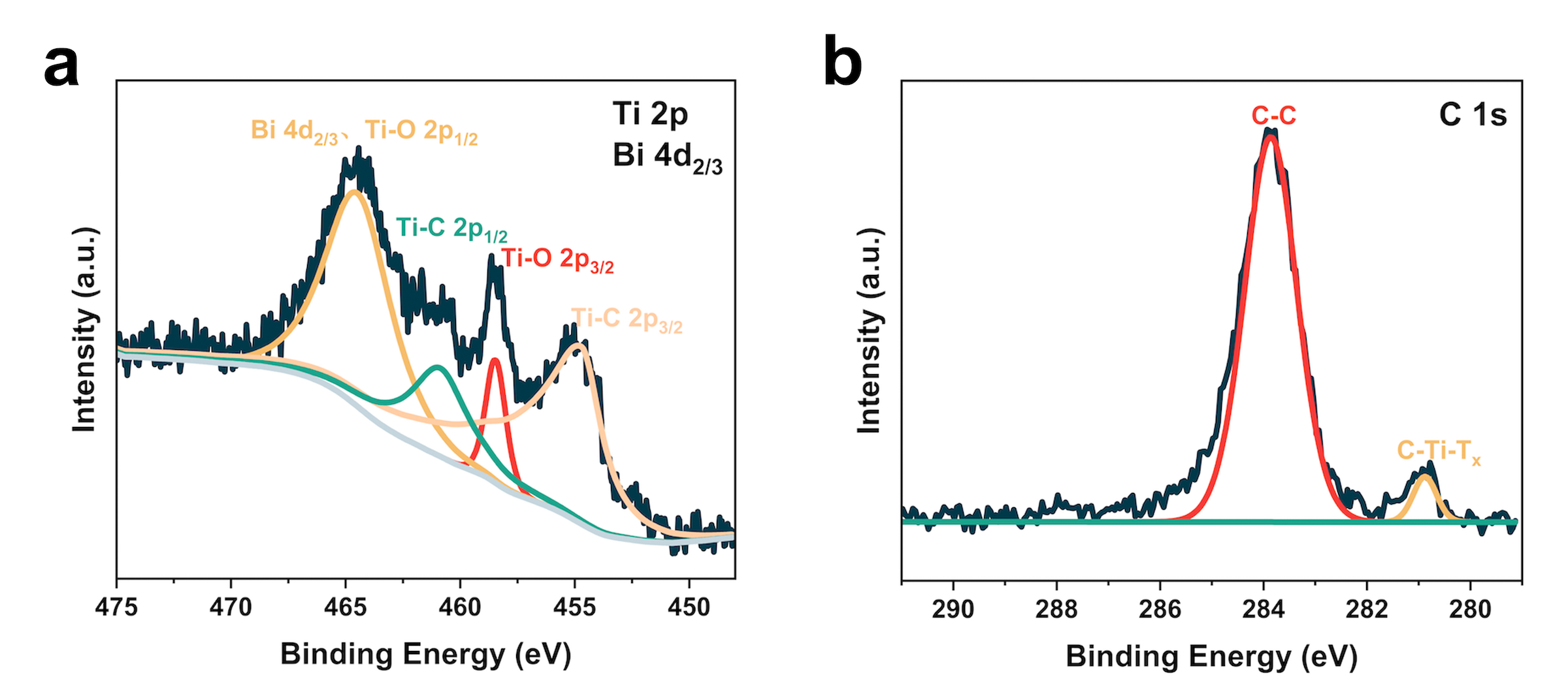
**

**Fig. S2** XPS results of Bi_2_S_3_/Ti_3_C_2_ NHs for the regions of interest.

**
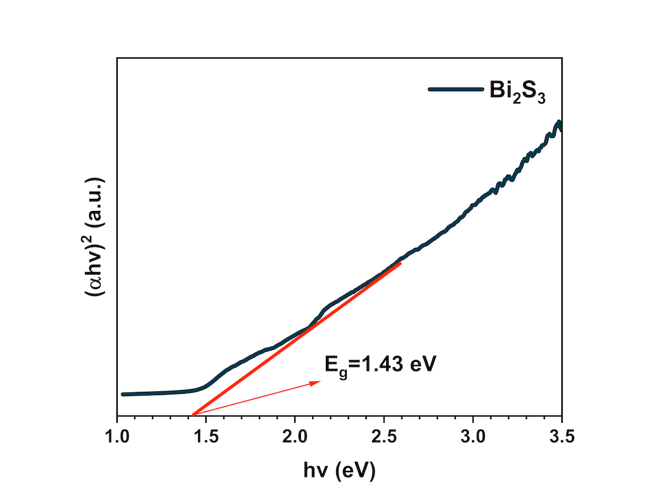
**

**Fig. S3** Tauc plot diagram of Bi_2_S_3_ NPs.

**
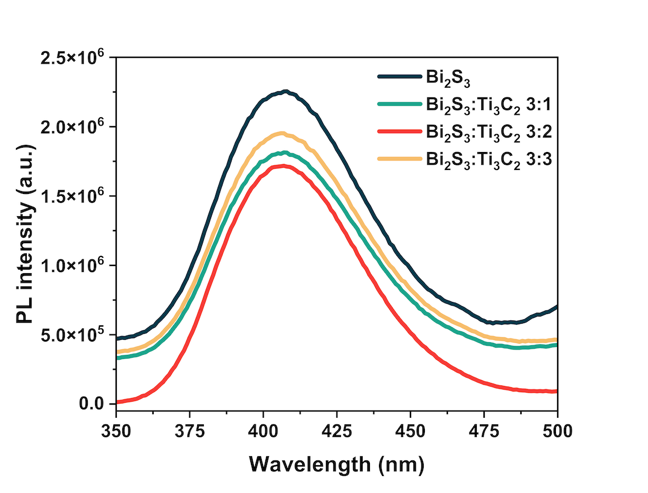
**

**Fig. S4** PL spectra of Bi_2_S_3_ NPs and Bi_2_S_3_/Ti_3_C_2_ NHs with different ratios.

**
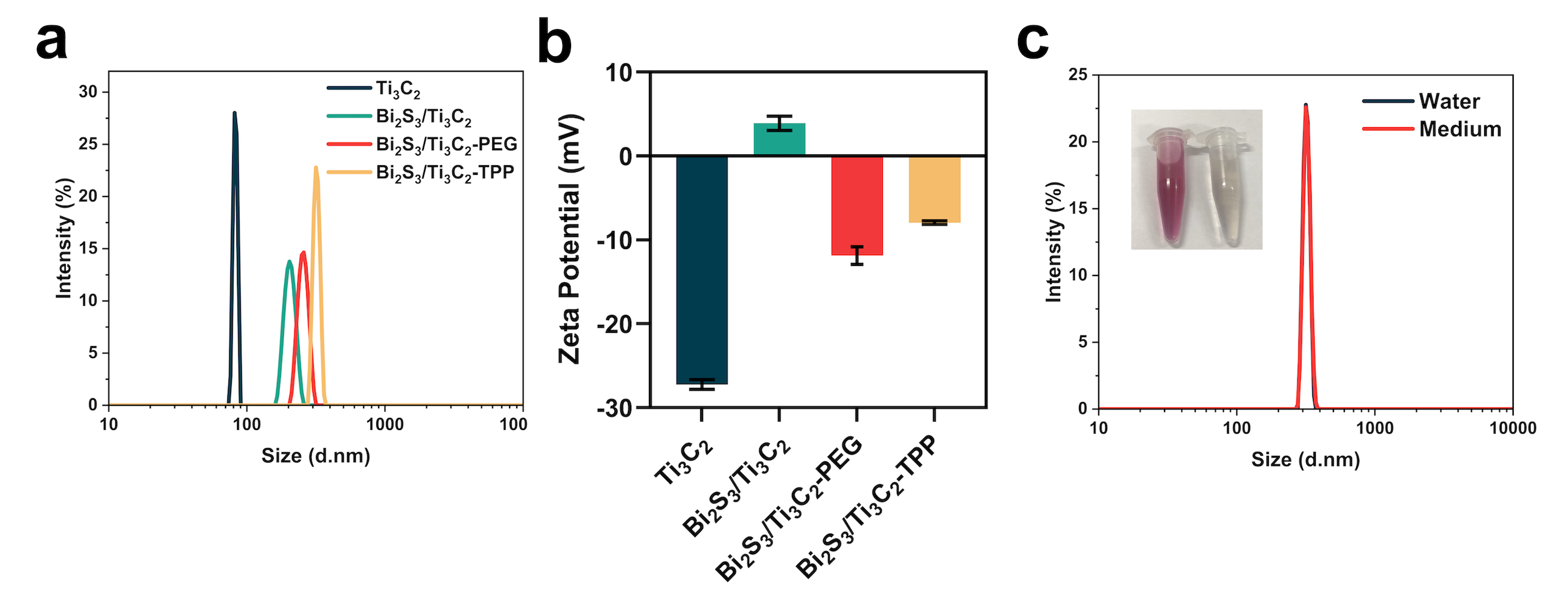
**

**Fig. S5 a** DLS and **b** Zeta potential of Ti_3_C_2_, Bi_2_S_3_/Ti_3_C_2_, Bi_2_S_3_/Ti_3_C_2_-PEG, and Bi_2_S_3_/Ti_3_C_2_-TPP. **c** Size distributions of Bi_2_S_3_/Ti_3_C_2_-TPP in water and DMEM; inset: corresponding image of Bi_2_S_3_/Ti_3_C_2_-TPP in DI and DMEM after 48 h storage.


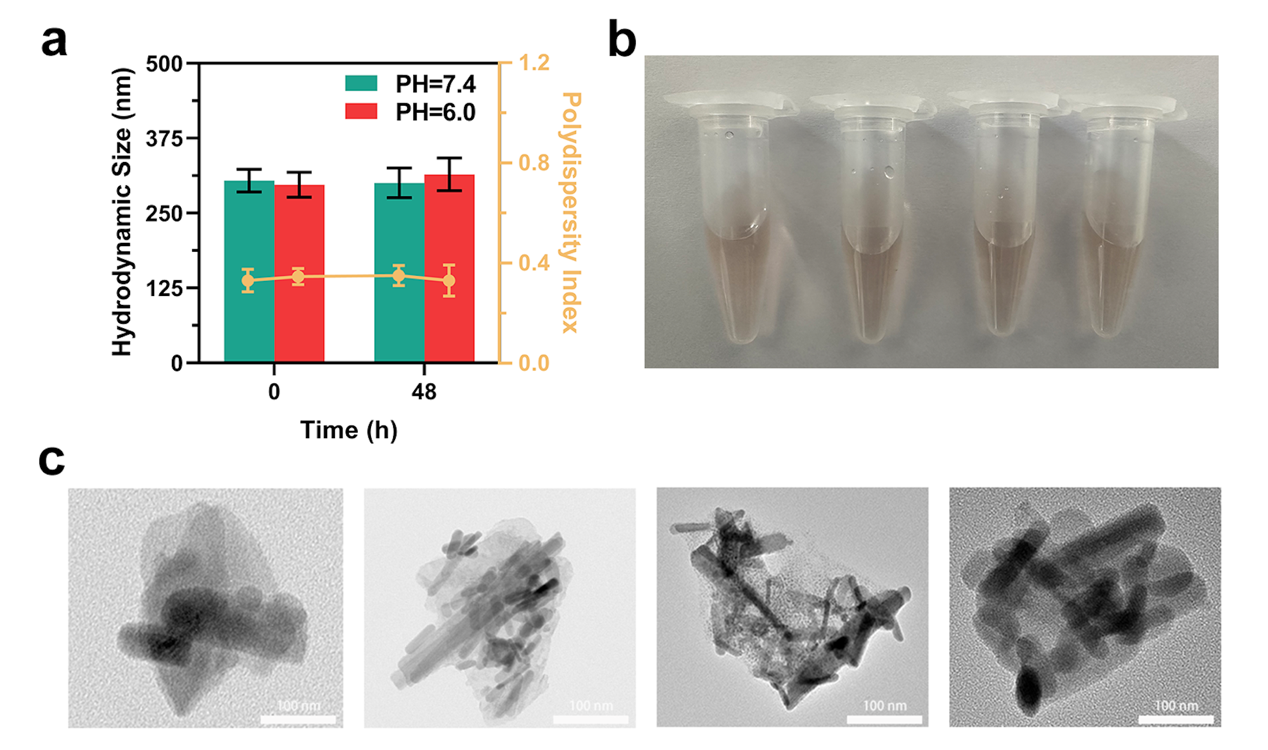


**Fig. S6 a** Hydrodynamic size and polydispersity index, and the corresponding **b** digital and **c** TEM images of Bi_2_S_3_/Ti_3_C_2_-TPP under different conditions. The sequence of the experimental group in pictures **b** and **c** from left to right is consistent with that in picture **a**.

**
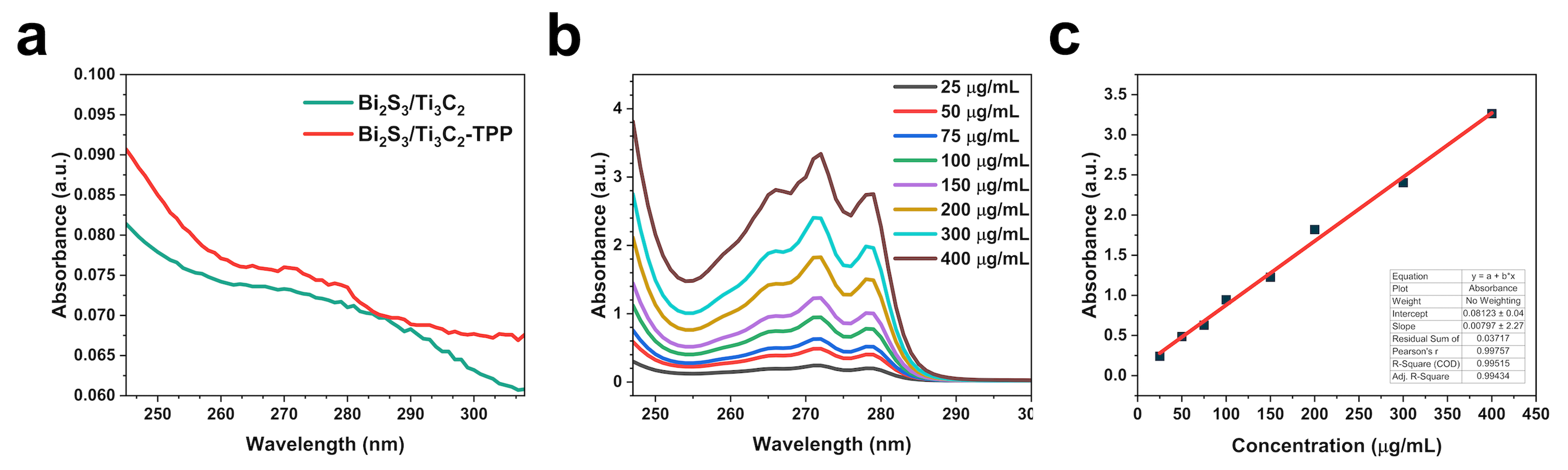
**

**Fig. S7** UV-vis absorption patterns of **a** Bi_2_S_3_/Ti_3_C_2_ and Bi_2_S_3_/Ti_3_C_2_-TPP, and **b** TPP aqueous solutions with various concentrations. **c** The standard curve of TPP concentrations (y = 0.0797x + 0.08123, R^2^ = 0.994).

**
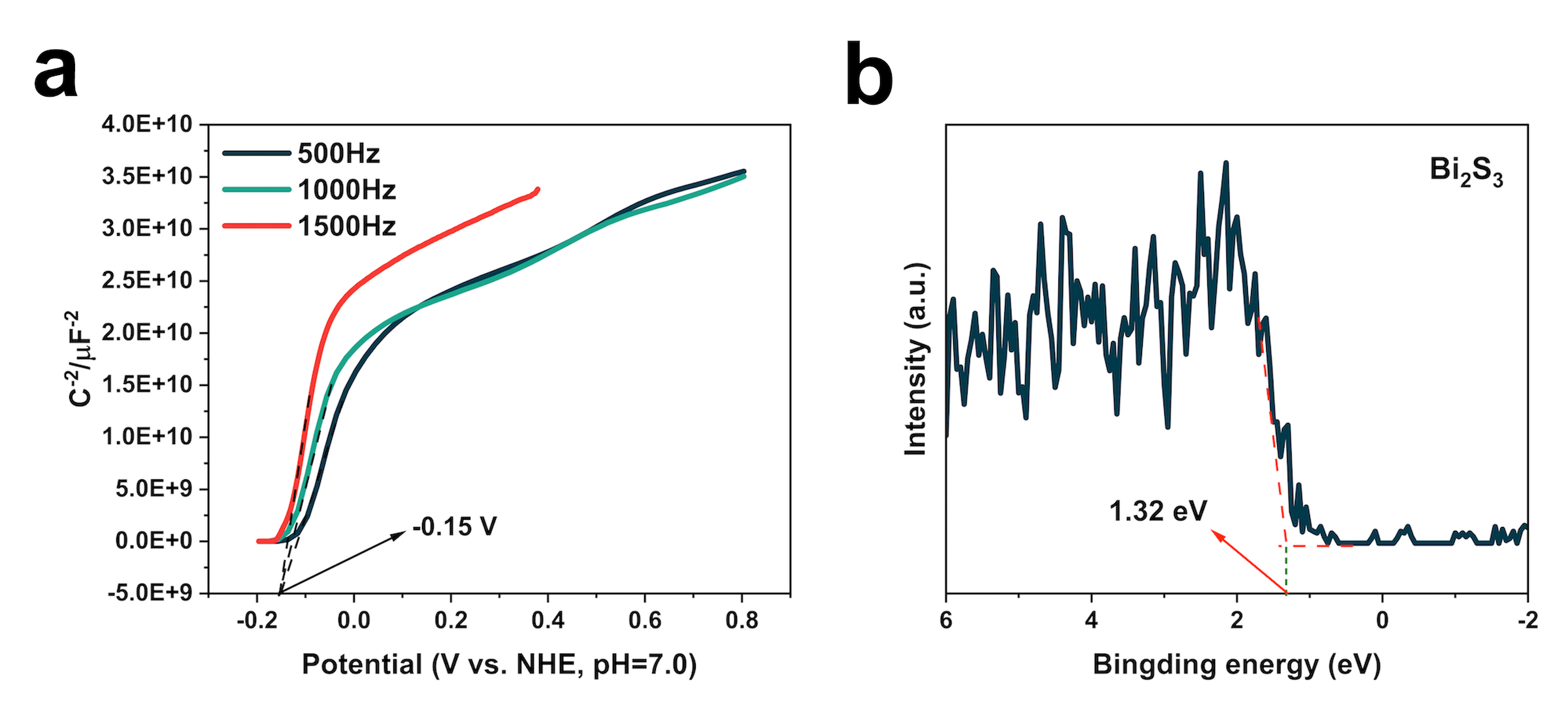
**

**Fig. S8 a** Mott-Schottky plot of Bi_2_S_3_. **b** XPS spectrum of VB in Bi_2_S_3_.

**
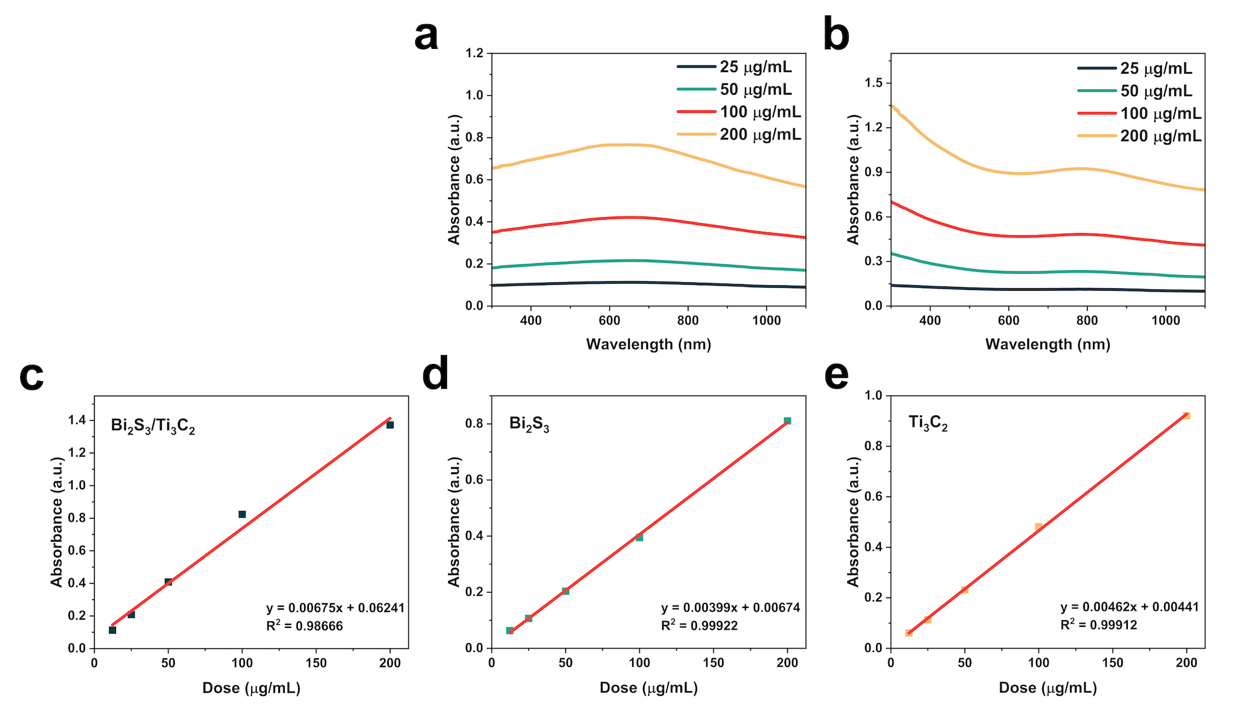
**

**Fig. S9** The UV–vis absorption spectra of **a** Bi_2_S_3_ and **b** Ti_3_C_2_ with various concentrations. The absorptions of **c** Bi_2_S_3_/Ti_3_C_2_, **d** Bi_2_S_3_, and **e** Ti_3_C_2_ aqueous suspensions with various concentrations at 808 nm.

**
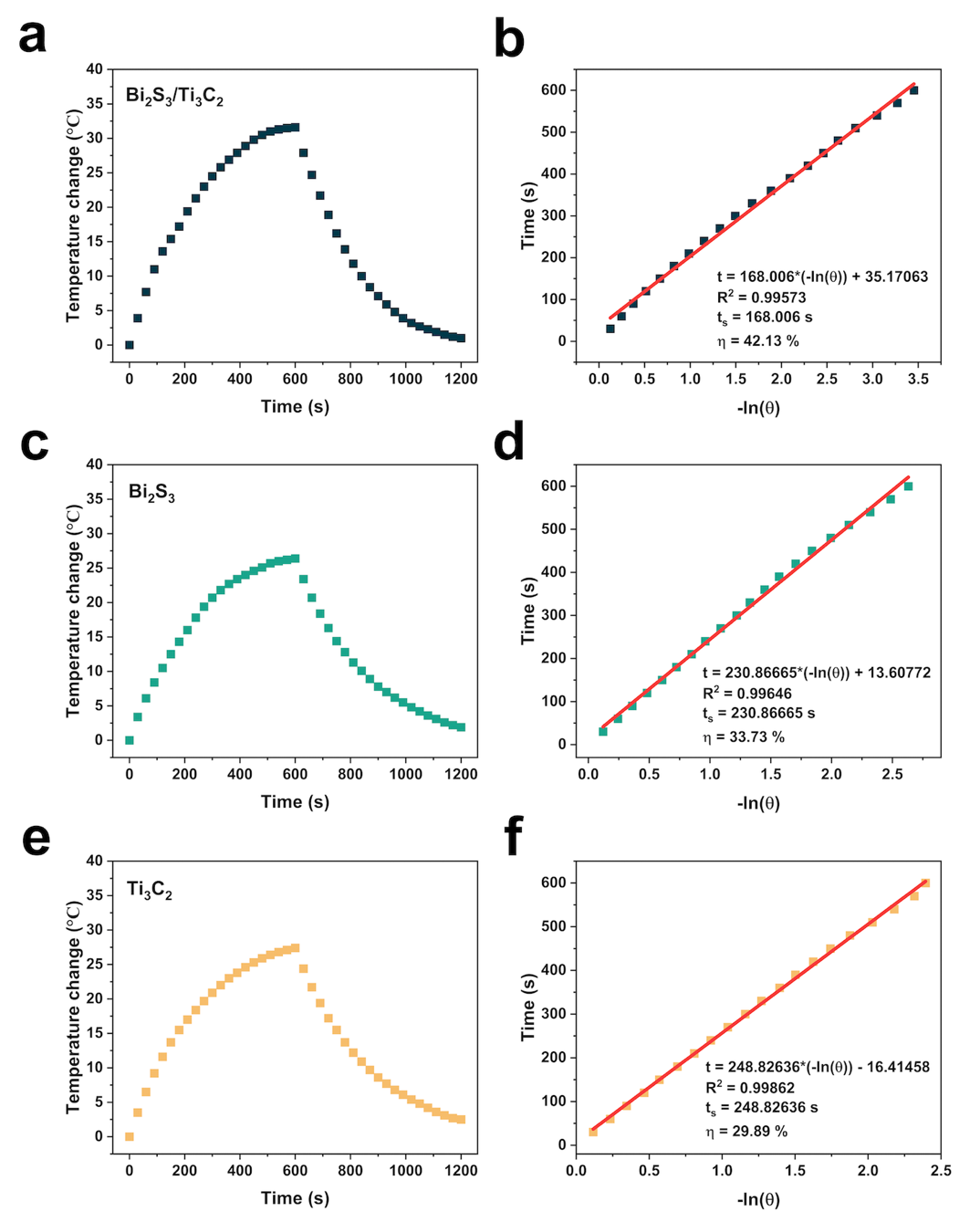
**

**Fig. S10** Heating and cooling profiles of **a** Bi_2_S_3_/Ti_3_C_2_, **c** Bi_2_S_3_, and **e** Ti_3_C_2_ (100 µg mL^-1^) under 808 nm laser irradiation (1 W cm^-2^, 10 min). Plot and linear fit of time *vs.* negative log of increased temperature for **b** Bi_2_S_3_/Ti_3_C_2_, **d** Bi_2_S_3_, and **f** Ti_3_C_2_ cooling rates.

**
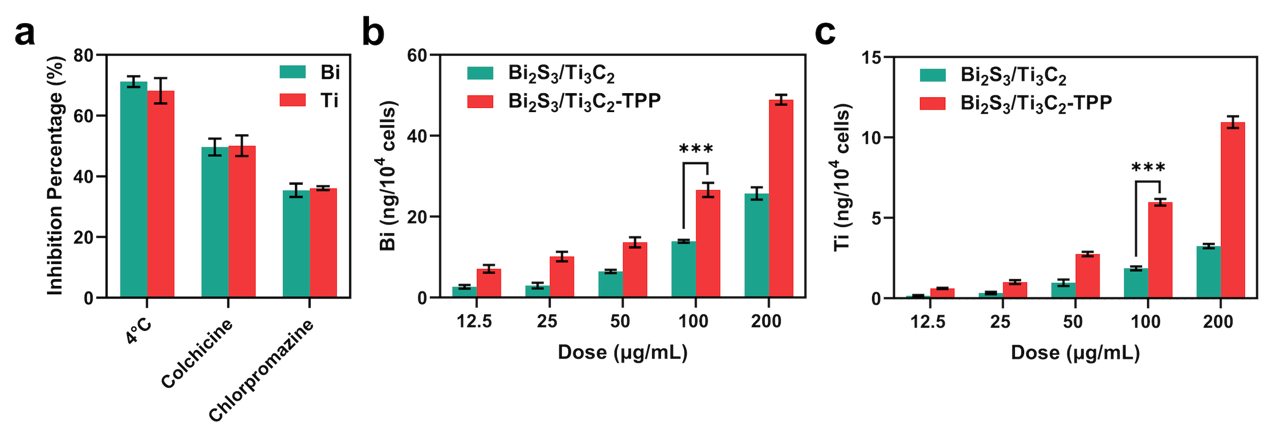
**

**Fig. S11 a** The inhibition effect of low temperature (4 ℃), colchicine, and CPZ on the cellular uptake of Bi_2_S_3_/Ti_3_C_2_-TPP. The contents of **b** Bi and **c** Ti in U251 cells incubated with Bi_2_S_3_/Ti_3_C_2_-TPP or Bi_2_S_3_/Ti_3_C_2_ measured by ICP-MS; ****P* < 0.001.

**
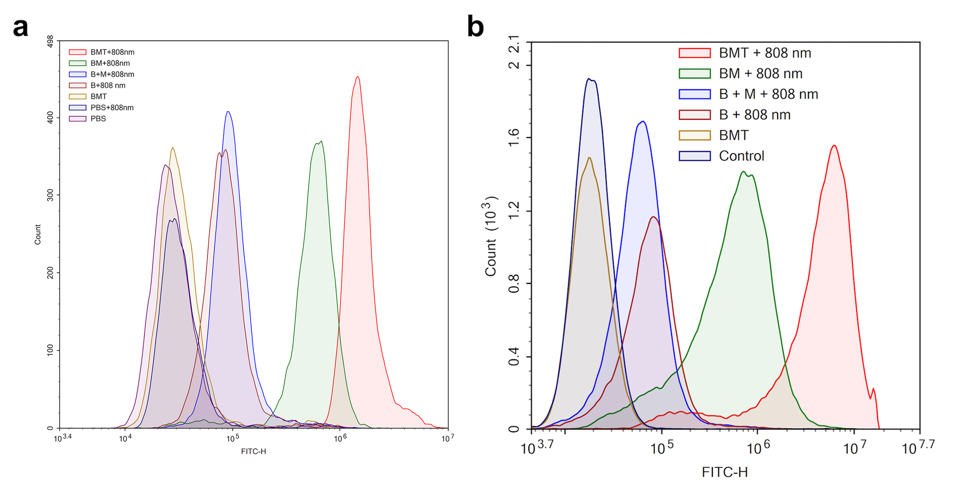
**

**Fig. S12 a** Flow cytometry analysis of JC-1-based MMP change in U251 cells. **b** DCF-based cellular ROS level of U251 cells analyzed by flow cytometry. B-Bi_2_S_3_, M-Ti_3_C_2_, T-TPP, sample concentration - 100 µg mL^-1^, and NIR - an 808 nm laser, 1 W cm^-2^, 10 min.


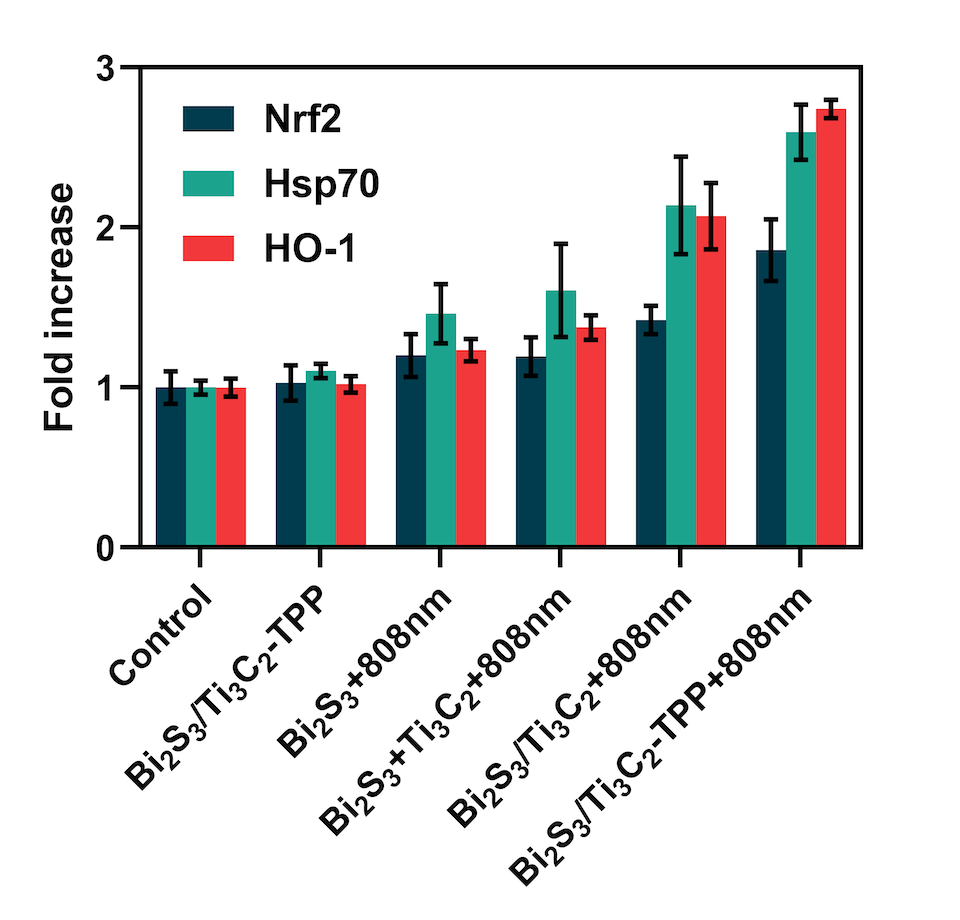


**Fig. S13** Quantification of Nrf2, Hsp70, and HO-1 expressions in U251 cells following various treatments; the intensities of Nrf2, Hsp70, and HO-1 were normalized to those of β-actin using Image J software.


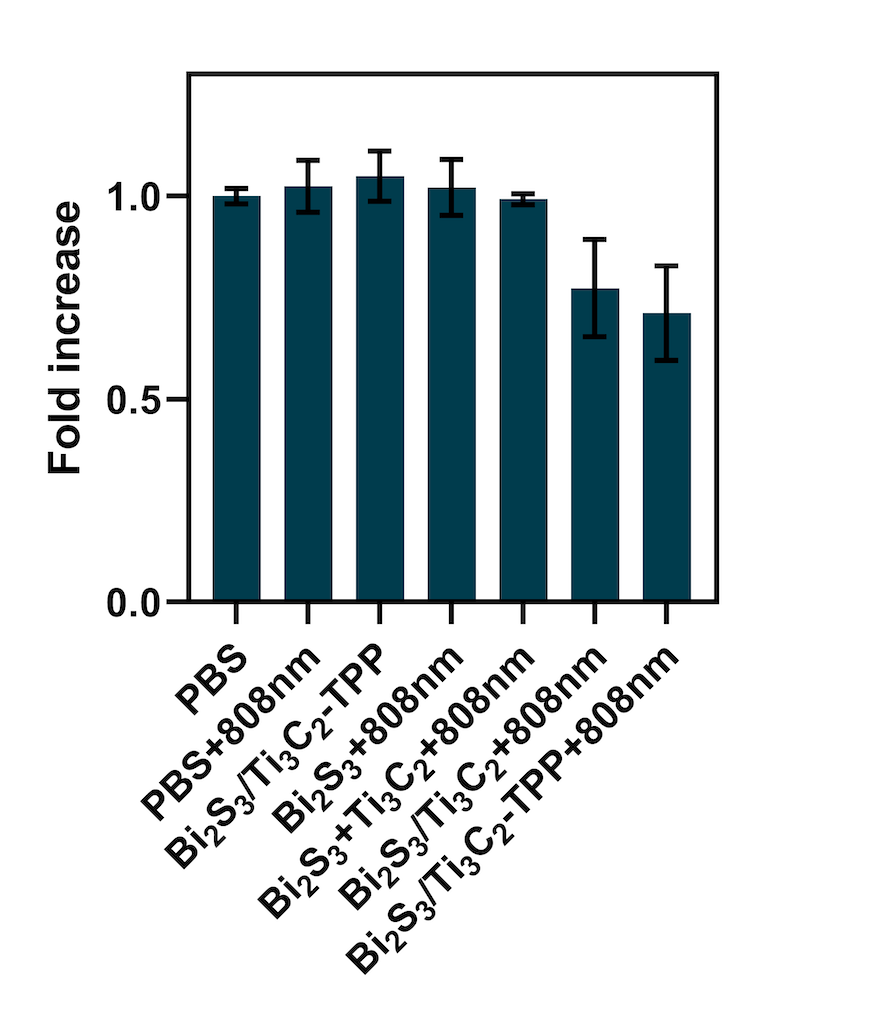


**Fig. S14** Quantification of HIF-1α expressions in tumors after various treatments, the intensities of HIF-1α were standardized to those of β-actin using Image J software.


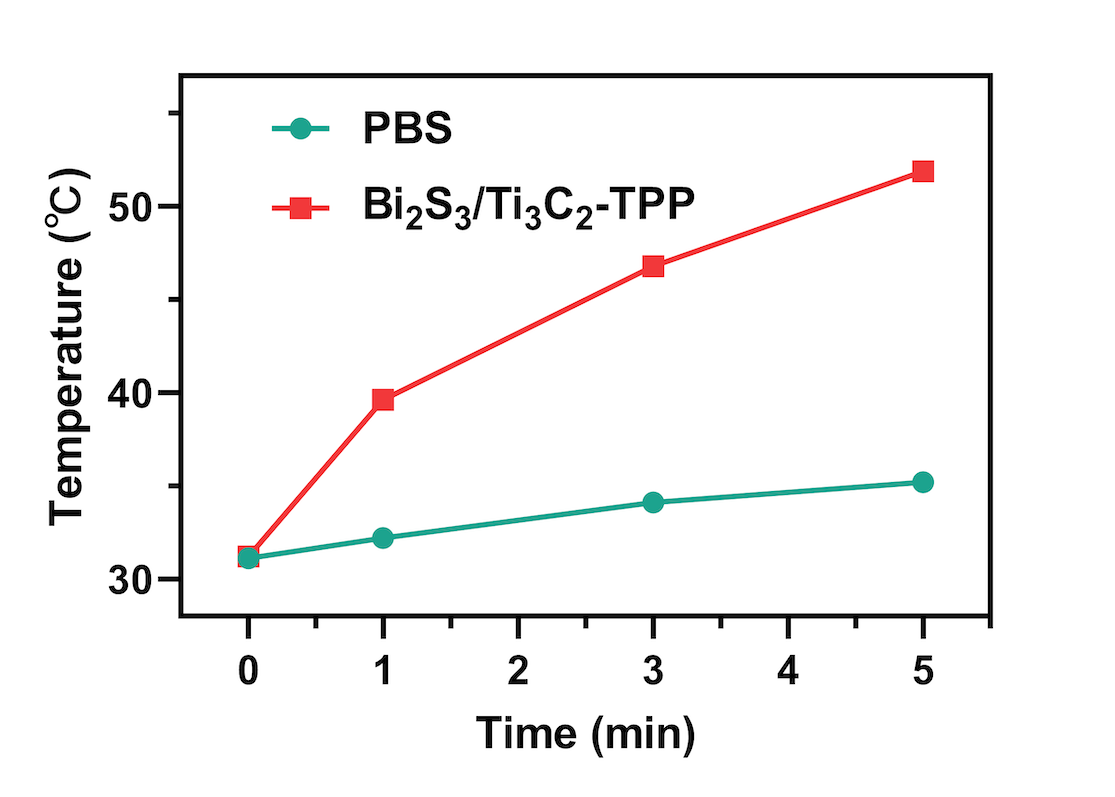


**Fig. S15** Plots of temperature values of U251 tumor-bearing mice after being injected with PBS (pH=7.4, 10 mM) or Bi_2_S_3_/Ti_3_C_2_-TPP (20 mg kg^-1^).
